# Blocking TRIM47-mediated HNF4*α* degradation suppresses hepatocellular carcinoma progression

**DOI:** 10.1016/j.apsb.2025.10.045

**Published:** 2025-11-01

**Authors:** Huanyu Hong, Mengchao Xiao, Hui Qian, Siqi Tan, Sihan Wu, Fang Liu, Xialu Hong, Shuqing Liu, Chenhong Ding, Keqi Wang, Weifen Xie, Xin Zhang

**Affiliations:** a Department of Gastroenterology, Changzheng Hospital, Naval Medical University, Shanghai 200003, China; b Department of Gastroenterology, Shanghai East Hospital, School of Medicine, Tongji University, Shanghai 200092, China; c Department of Gastroenterology, Jiangnan University Medical Center (Wuxi No.2 People’s Hospital), Wuxi 214002, China

**Keywords:** Hepatocellular carcinoma, Protein–protein interactions, TRIM47, HNF4*α*, Ubiquitination, Virtual screening, Small molecule, CZ-2401

## Abstract

Previous studies have highlighted the downregulation of hepatocyte nuclear factor 4alpha (HNF4*α*) as a critical event in the pathogenesis of HCC. However, the mechanism of its degradation in HCC remains unclear. Tripartite motif 47 (TRIM47), a typical E3 ubiquitin ligase of the TRIM family, has been implicated in various tumors, yet its specific role in HCC progression is not fully elucidated. In this study, HNF4*α* was identified as a potential target of TRIM47 by using co-immunoprecipitation (Co-IP) combined with mass spectrometry analysis. TRIM47 facilitates the degradation of HNF4*α* by mediating K48-linked ubiquitination at lysine 470. Abrogation of HNF4*α* ubiquitination attenuated the promoting effect of TRIM47 on HCC malignancy. Molecular docking studies and Co-IP experiments revealed that K342, W349, and E353 of HNF4*α*, along with K534 and K600 of TRIM47, are crucial for their interaction. A small molecule, CZ-2401, was selected as a potent inhibitor of the TRIM47–HNF4*α* interaction through virtual screening and pharmacological activity validation. CZ-2401 effectively stabilizes HNF4*α* protein in HCC cells and ameliorates TRIM47-driven HCC progression *in vivo.* Taken together, our research elucidates that targeting TRIM47–HNF4*α* interaction is a potential therapeutic strategy for HCC, and identifies CZ-2401 as a potent inhibitor of HNF4*α* degradation and a promising candidate for HCC therapy.

## Graphical abstract

TRIM47 binds to HNF4*α* and facilitates its ubiquitination and degradation in HCC progression. CZ-2401 is a potent inhibitor of TRIM47–HNF4*α* interaction and a promising candidate for HCC therapy.

**Figure:**
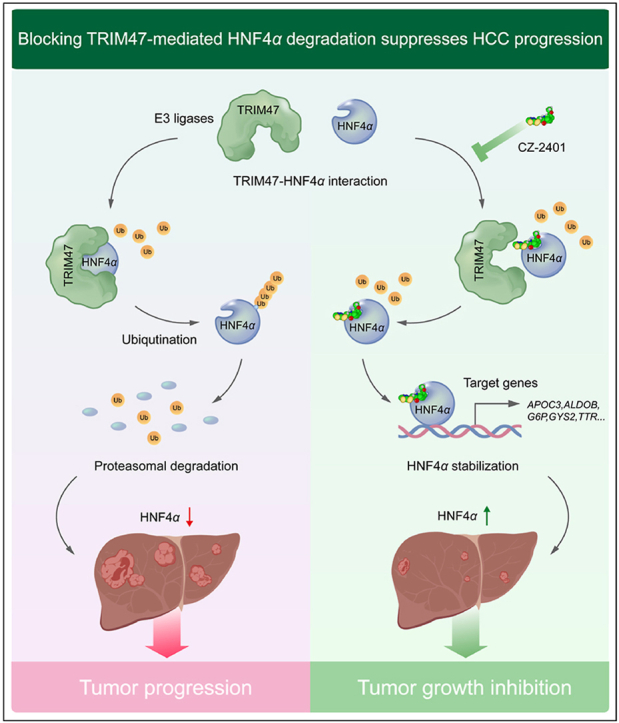

## 1 Introduction

Hepatocellular carcinoma (HCC) ranks as the sixth most common cancer and the third leading cause of cancer-related deaths worldwide, with a poor average five-year survival rate according to 2020 statistics^1^. Although systemic anti-cancer therapies, including chemotherapy, specific targeted drug therapy, and immunotherapy, have improved the quality of life and overall survival of HCC patients, their therapeutic outcomes remain unsatisfactory^2^^,^^3^. Thus, it is urgent to further elucidate the mechanisms underlying the onset and progression of HCC to inform the development of targeted therapeutics^4^.

The ubiquitin–proteasome system is integral to the degradation of the majority of cellular proteins^5^. Dysregulation of the ubiquitin system can significantly impact the activity and degradation of oncogenic and tumor suppressor proteins, resulting in alterations in key biological processes such as the cell cycle, invasion and metastasis, cancer stem cell traits, and immune microenvironment dynamics, which suggests that targeting the ubiquitin–proteasome degradation pathway holds potential as a therapeutic strategy for cancer^6^^,^^7^. Tripartite motif 47 (TRIM47) is a highly conserved E3 ubiquitin-protein ligase, characterized by a canonical N-terminal TRIM domain and a C-terminal PRY-SPRY domain^8^. It plays a crucial role in mediating protein–protein interactions and substrate recognition within the ubiquitin network^9^. Previous studies have shown that TRIM47 promotes the proliferation of pancreatic, glioma, renal, and breast cancer cells by respectively affecting the degradation of FBP1, FOXO1, p53, and PKCε^10^, ^11^, ^12^, ^13^. These findings underscore TRIM47’s ability to recognize a diverse range of substrates and catalyze various types of ubiquitination, thereby influencing tumor progression. A recent study demonstrates that TRIM47 drives HCC progression through suppressing ferroptosis by inducing K48-linked ubiquitination and proteasomal degradation of CDO1^14^. Moreover, TRIM47 protects SNAI1 from degradation to promote EMT and metastasis in HCC^15^. These findings position TRIM47 as a promising therapeutic target in HCC. However, the roles and underlying mechanisms of TRIM47 in HCC remain to be further clarified.

Hepatocyte nuclear factor 4alpha (HNF4*α*) is a conserved, ligand-dependent transcription factor within the nuclear receptor superfamily, predominantly expressed in mature hepatocytes^16^. It plays a critical role in liver development, hepatocyte maturation and differentiation, and the maintenance of essential hepatocyte functions^17^. Downregulation of HNF4*α* is closely associated with the onset and progression of chronic liver diseases, the malignant transformation of hepatocytes, and the invasion and metastasis of liver cancer, making it a promising therapeutic target for various liver diseases^18^^,^^19^. Our previous studies showed that HNF4*α* delivery reverses the malignant phenotypes of HCC by inducing the redifferentiation of HCC cells toward hepatocytes^20^. It has been reported that the levels and activity of HNF4*α* can be regulated by various protein post-translational modifications, including acetylation, phosphorylation, methylation, and ubiquitination^21^, ^22^, ^23^, ^24^. Our recent studies have demonstrated that blocking the ubiquitination-mediated degradation of HNF4*α*, catalyzed by the TRIM8-TRIB3 E3 ligase complex, ameliorates non-alcoholic fatty liver disease (NAFLD) progression in mice, providing a novel strategy for treating NAFLD and even other liver diseases by stabilizing HNF4*α* protein^25^. However, the diverse mechanisms underlying HNF4*α* degradation in HCC progression require further investigation.

In this study, we found that the E3 ligase TRIM47 contributes to HCC progression by binding HNF4*α* and facilitating its ubiquitination and degradation, and we hypothesize that pharmacologic inhibitors disrupting the TRIM47–HNF4*α* interaction could provide therapeutic benefits in HCC. To date, small molecules with high selectivity for binding to proteins, obtained through structure-based and phenotypic screening in drug design, have shown improved biological activity and reduced side effects, such as the MDM2 inhibitor APG-115, which destabilizes MDM2–p53 complexes and restores p53 activity, showing remarkable efficacy in treating chronic lymphocytic leukemia^26^. Herein, using molecular docking-based virtual screening, we identified a small molecule compound as an effective inhibitor of the TRIM47–HNF4*α* interaction, which can significantly suppress the malignant phenotype in liver cancer models, representing an ideal strategy toward the development of targeted therapeutics to treat HCC by stabilizing HNF4*α*.

## 2 Materials and methods

### 2.1 Human liver and clinical specimens

In this study, all human liver specimens were diagnosed as HCC *via* postoperative pathology. HCC tissue samples were collected from the Eastern Hepatobiliary Surgery Hospital and Changzheng Hospital, Naval Medical University in Shanghai, China. Written informed consent was obtained from all participants before sample collection. All procedures involving the collection and utilization of human samples were approved by the Ethics Committee of Naval Medical University (Approval No. 82273096, China) and complied with the principles of the Declaration of Helsinki.

### 2.2 Virus

To achieve TRIM47 overexpression, HEK-293T cells were co-transfected with the TRIM47 lentiviral plasmid, the packaging plasmid psPAX2, and the envelope plasmid pMD2.G using Lipofectamine 2000 (11668019, Invitrogen, Carlsbad, USA). All plasmids were sourced from YouBio (Changsha, China). After 48 h, the medium containing lentivirus was collected, and the viral particles were concentrated and stored at −80 °C for later use. Recombinant adenoviruses AdGFP and AdHNF4*α* were established in our lab, while AdHNF4*α* (K470R) was purchased from WzBio (Shandong, China).

### 2.3 Cell lines and cell culture

Human hepatocellular carcinoma cell lines (Huh7, Hep3B, and MHCC-L) and HEK293T cells were procured from the Type Culture Collection of the Chinese Academy of Sciences in Shanghai, China. Authentication of these cells was conducted *via* Short Tandem Repeat (STR) analysis, and mycoplasma contamination was confirmed as negative using the Mycoplasma Detection Kit (MP0050, Sigma–Aldrich, MO, USA). TRIM47 knockout cells were generated in the Huh7 background utilizing the CRISPR-Cas9 system from Cyagen Biosciences Inc. (Guangzhou, China). All cells were cultured in DMEM (CM10017, Macgene, Beijing, China) supplemented with 10% fetal bovine serum (FBS, Gibco, CA, USA) and incubated at 37 °C in a humidified atmosphere with 5% CO_2_.

### 2.4 qRT-PCR

Total RNA was extracted from cultured HCC cells or tissues using Trizol reagent (Takara, Shiga, Japan), and 2000 ng of RNA was reverse transcribed using the Evo M-MLV RT Kit (Promega, WI, USA). Subsequent qRT-PCR analysis was performed with SYBR Green detection. The expression of target transcripts was normalized to *β*-actin, and relative gene expression was calculated using the 2^–ΔΔ*C*t^ method. The primers used in this study are detailed in Supporting Information Table S1.

### 2.5 Western blotting (WB)

HCC tissues or cells were lysed with 1% SDS lysis buffer supplemented with protease and phosphatase inhibitors (11697498001, Roche, Basel, Switzerland). Protein concentrations were quantified using the BCA Protein Assay Kit (P0010, Beyotime, Shanghai, China). Equal amounts of protein were separated by 10% SDS-PAGE and transferred onto methanol-activated nitrocellulose membranes. Membranes were blocked in PBS buffer containing 0.1% Tween 20 and 5% milk at room temperature for 1–3 h, followed by incubation with primary antibodies at 4 °C overnight. After incubation with secondary antibodies in the dark for 1 h, protein signals were detected and quantified using the Odyssey infrared imaging system (LI-COR Biosciences, Lincoln, USA). The antibodies used in this study are shown in Supporting Information Table S2.

### 2.6 RNA interference and plasmid constructs

siRNAs targeting human *TRIM47* (GenBank: NM_033452.3) and human *HNF4A* (GenBank: NM_000457.6) were purchased from GenePharma (Shanghai, China), and plasmids were constructed by YouBio (Changsha, China). Transfections were performed using Lipofectamine 2000 according to the manufacturer’s protocols. The siRNA sequences are listed in Supporting Information Table S3. Full-length TRIM47 (amino acids 1–638) and its truncations and mutations, including ΔRING (amino acids Δ1–85), 1–216 (amino acids 1–216), 217–638 (amino acids 217–638), Δ478–605 (amino acids Δ478–605), K534A, K600A and K2A (K534A/K600A), were cloned into the pcDNA3.0-Flag vector using seamless cloning. Full-length HNF4*α* (amino acids 1–474) and its truncations and mutations of HNF4*α*, including 1–163 (amino acids 1–163), 1–368 (amino acids 1–368), 164–474 (amino acids 164–474), K342A, W349A, E353A, 3A (K342A/W349A/E353A) and K470R, were similarly cloned into the pcDNA3.0-V5 vector. Plasmids expressing ubiquitin and ubiquitin K48O, K48R, and K63R were created by YouBio (Changsha, China) using the pcDNA3.0-HA vector, with the pcDNA3.0 plasmid serving as a control. *TRIM47* was also cloned into the pcDNA3.0-Flag vector.

### 2.7 Proliferation, migration, invasion, and colony formation assays

Huh7 cells infected with PCDH or LV-TRIM47, and wild-type Huh7 cells or TRIM47-KO Huh7 cells were plated at a density of 3 × 10^3^ cells per well in a 96-well plate, respectively. Hep3B cells transfected with siNC or siTRIM47-1 or siTRIM47-2 for 24 h were seeded into the 96-well plates at 3 × 10^3^ cells per well. The cells were cultured in DMEM supplemented with 10% FBS, while Huh7-PCDH and Huh7-LV-TRIM47 cells were cultured in DMEM supplemented with 5% FBS, with media changes performed every three days for 6 days. Hep3B cells infected with PCDH or LV-TRIM47 cells were plated at a density of 3 × 10^3^ cells per well in a 96-well plate and treated with CZ-2401 or DMSO at 10 μmol/L for 6 days, with medium replenished every other day. Metabolic activity of these cells was assessed daily using the CCK-8 kit (Dojindo Laboratories, Kumamoto, Japan). After an incubation of 1h at 37 °C, the absorbance was measured at 450 nm using a microplate reader (BioTek Instruments, Winooski, VT, USA).

Migration and invasion were evaluated using Transwell chambers with or without Matrigel (BD Biosciences, San Jose, CA, USA). Serum-starved Huh7 or Hep3B cells (3 × 10^4^) in DMEM were placed in the upper chamber, while the lower chamber contained 600 μL DMEM with 10% FBS. After incubation at 37 °C for 48–72 h, cells were fixed with 4% paraformaldehyde and stained with 0.1% crystal violet. Five fields were photographed, and the cells were counted to estimate density, with the stained area measured using Image-Pro Plus 6.0 software.

For the colony formation experiments, the cells were detached with trypsin and transferred to a 6-well plate at 1 × 10^3^ cells per well. Following a 2-week incubation, the plates were washed with PBS and fixed in 4% paraformaldehyde for 20 min and then stained with 0.005% crystal violet. The images were scanned and analyzed.

### 2.8 Flow cytometry (FACS) for apoptosis analysis and intracellular Ki-67 staining

For flow cytometry analysis, Huh7 and Hep3B cells seeded at 5 × 10^5^ per well were treated with CZ-2401 at 10 or 25 μmol/L for 24 h. Cells were washed twice with pre-cold BioLegend Cell Staining Buffer (420201, BioLegend, San Diego, CA, USA), and further stained with Fluorochrome-labeled Annexin V and Propidium Iodide Solution (PI) at 4 °C in the dark for 20 min according to the manufacturer’s protocol (640915, BioLegend). Early apoptotic (Annexin V^+^/PI^−^) and late apoptotic (Annexin V^+^/PI^+^) populations were analyzed using a flow cytometer (Cytoflex S, Beckman, USA).

To perform intracellular Ki-67 staining, Huh7 and Hep3B cells with the indicated treatment were collected and incubated with permeabilization buffer (424401, BioLegend) at 4 °C in the dark for 1 h. The cells were then incubated with the fluorescently labeled Ki-67 antibody according to the manufacturer’s protocol. After intracellular staining for another 1 h, the cells were washed, centrifuged, and resuspended in PBS. The Ki-67-positive population was evaluated by flow cytometry (Cytoflex S, Beckman).

### 2.9 Animals and treatment

Adult male BALB/c nude mice (4–6 weeks old) were purchased from Shanghai Bikai and maintained under specific pathogen-free conditions with a 12 h light/dark cycle at 20–25 °C. All animal experimental protocols were approved by the Animal Care and Use Committee of Naval Medical University and conducted in accordance with the National Institutes of Health guidelines for the care and use of laboratory animals.

To evaluate the effect of TRIM47 on HCC progression, twenty mice were randomly assigned to two groups, with Huh7 cells infected with TRIM47 lentivirus (2 × 10^6^ cells in 100 μL DMEM) subcutaneously injected into the right flank of each group (*n* = 10). To evaluate the effect of HNF4*α* on TRIM47-driving HCC growth, forty mice were randomly divided into four groups, and Huh7 cells infected with TRIM47 or control lentivirus were injected as described above (*n* = 10 per group). Twelve days post-injection, these groups received intratumoral injections of 2 × 10^9^ pfu AdGFP, AdHNF4*α*, or AdHNF4*α* (K470) every two days (*n* = 6 per group).

To detect the therapeutic effect of CZ-2401 on HCC, forty mice were divided into four groups and received Hep3B cells infected with TRIM47 or control lentivirus (*n* = 10 per group). Twenty-seven days after subcutaneous injection, these groups were treated with intraperitoneal injections of CZ-2401 at a dose of 8 mg/kg daily (*n* = 7 per group). Tumor volume was calculated as Eq. (1):

(1) Tumor column = Width^2^ × Length × 0.5

### 2.10 Immunohistochemistry (IHC)

Tissue paraffin sections of approximately 3–5 μm thickness were deparaffinized in xylene and rehydrated through graded alcohols. Endogenous peroxidases were inactivated with 0.3% H_2_O_2_ for 10–15 min, followed by antigen retrieval using EDTA. The sections were blocked with 10% goat serum at 37 °C for 1 h and then incubated with primary antibodies at 4 °C overnight. After three washes with PBST, the sections were incubated with corresponding secondary antibodies for 1 h, followed by visualization using the EnVision Detection Rabbit/Mouse Kit (GK500710, Genentech, South San Francisco, CA, USA). The antibodies used for IHC are listed in Table S2.

### 2.11 Mass spectrometry analysis

MHCC-L cells transfected with Flag-TRIM47 or Flag-vector were treated with MG132 (HY-13259, MedChemExpress) for 6 h, lysed in immunoprecipitation (IP) buffer, and subjected to IP with anti-Flag beads at 4 °C overnight. The beads were extensively washed with wash buffer (20 mmol/L Tris-HCl, pH 8.0, 150 mmol/L NaCl, 0.5% NP40, 0.5% Triton X-100, 10% glycerol) followed by PBS. Bound proteins were then digested with trypsin, and the resulting peptides were analyzed by mass spectrometry using a Q Exactive HF-X mass spectrometer (Thermo Scientific, Waltham, MA, USA). The mass spectrometry proteomics data have been deposited to the ProteomeXchange Consortium (https://proteomecentral. proteome exchange.org) *via* the iProX partner repository with the dataset identifiers PXD062446^27^^,^^28^. The complete dataset can be publicly accessible through this identifier.

### 2.12 DUOlink proximity ligation assay (PLA)

Huh7 cells transfected with TRIM47 and HNF4*α* for 48 h were cultured on coverslips, fixed with 4% PFA for 15 min at room temperature, washed twice with PBS, and blocked with blocking solution (DUO82007, Sigma–Aldrich) at 37 °C for 1 h. Primary antibodies were incubated overnight at 4 °C. After three washes with Buffer A (DUO82049, Sigma–Aldrich), cells were incubated with 1:5-diluted PLUS and MINUS Duolink PLA probes (DUO92002/DUO92004, Sigma–Aldrich) at 37 °C for 1 h. Following three times with Buffer A, the samples were treated with 1 U/μL T4 DNA ligase (DUO92013, Sigma–Aldrich; 1:5 in ligation buffer) at 37 °C for 60 min, and amplified with 5 U/μL DNA polymerase (DUO92013, Sigma–Aldrich; 1:5 in polymerase buffer) at 37 °C for 120 min. Samples were mounted with DAPI-containing DUOlink mounting medium, and fluorescence images were acquired on a Leica TCS SP8 confocal microscope.

### 2.13 Dual-luciferase reporter assay

8 × 10^4^ Huh7 and Hep3B cells were seeded in 96-well plates and co-transfected with the HNF4*α* reporter plasmid pGL3-NINJ1-9p^29^, the control pRL-SV40 vector (Promega, Madison, USA), and either a TRIM47 overexpression or a control plasmid. Luciferase activities were assessed 24 to 48 h post-transfection using the Dual-Luciferase Reporter Assay System (Promega, Madison, USA), following the manufacturer’s protocol. Each experiment was conducted in triplicate. The functional validation experiments used a standardized 24 h CZ-2401 treatment protocol to reduce off-target effects.

### 2.14 Coimmunoprecipitation (Co-IP)

For exogenous Co-IP, HEK293T cells were transfected with plasmids encoding Flag-tagged TRIM47 or V5-tagged HNF4*α* and lysed using IP lysis buffer (Thermo Scientific, Waltham, USA) at 4 °C for 20–30 min. Cell debris was cleared by centrifugation at 12,000 rpm (CP 100NX, Eppendorf, Hamburg, Germany) for 15–30 min, and the supernatant was incubated with 10 μL anti-Flag (Sigma–Aldrich) or anti-V5 affinity gel (MilliporeSigma, Burlington, USA) overnight at 4 °C. The affinity gel was then separated by centrifugation at 3000 rpm (CP 100NX, Eppendorf, Hamburg, Germany) for 5 min and washed three times with wash buffer (20 mmol/L Tris-HCl, pH 7.4, 100 mmol/L NaCl, 0.5% NP40, 0.5% Triton-X100, 10% glycerol). Western blotting was then performed. For endogenous Co-IP, MHCC-L cells were lysed and incubated with 10 μL HNF4*α* antibody (Santa Cruz Biotechnology, Dallas, TX, USA) or 2 μL IgG and 10 μL protein G agarose at 4 °C overnight. The protein–antibody complexes were processed identically to the exogenous Co-IP protocol, and mass spectrometry was employed to identify proteins bound to the anti-Flag affinity gel.

### 2.15 Ubiquitination assay

Huh7 or Hep3B cells were transfected with the indicated plasmids, followed by treatment with 20 μmol/L MG132 for 6 h. Cells were lysed on ice with RIPA buffer containing 1% SDS and supplemented with protease and phosphatase inhibitors (11697498001, Roche, Basel, Switzerland) for 30 min. After sonication (amplitude: 50%; 10 s on/10 s off; 20 cycles), cell lysates were heated at 100 °C for 15 min and centrifuged at 12,000 rpm (CP 100NX, Eppendorf, Hamburg, Germany) for 15 min. The supernatants were diluted with RIPA buffer to reduce the SDS concentration and incubated overnight at 4 °C with pre-washed anti-V5 affinity gel (Merck Millipore, Burlington, USA) or anti-HNF4*α* antibody conjugated to protein A/G agarose resin. Beads were washed three times and boiled in 5 × loading buffer. Ubiquitination was then assessed by Western blotting.

### 2.16 Structure preparations of HNF4*α* and TRIM47

The three-dimensional structure of HNF4*α* was obtained from the PDB server (https://www.rcsb.org/structure/4IQR). The three-dimensional structure of TRIM47 was generated using the AlphaFold server (https://www.uniprot.org/uniprotkb/Q96LD4/entry). The 3D structure of HNF4*α* and TRIM47 was then protonated and optimized using the MOE plug-in “the Quickprep”.

### 2.17 Molecular docking

MOE plug-in “protein–protein docking” was used to study the interaction between HNF4*α* and TRIM47, where TRIM47 acts as the ligand and docks to the receptor protein 4IQR. Both 4IQR and TRIM47 were protonated under the AMBER10: EHT force field. The region of TRIM47 from S410-C631 was selected as the active region of molecular docking. During the docking process, the proteins were coarse-grained, and the fast Fourier transform was used to search for the interaction mode^30^. The coarse-grained model and the side chain of the contact residues were optimized through the Induced Fit scheme^31^. The docking poses were further optimized using energy minimization, and the binding energies were calculated using the GB/VI scoring function^32^. Finally, the top 100 docking poses were retained for further analysis.

### 2.18 In silico docking of small molecules

The HNF4*α* structure (Pose1) was protonated and optimized using the MOE plugin “Quickprep”. The key amino acids Trp349, Glu353, and Pro342 of the HNF4*α* structure were defined as the docking region. Under the AMBER10: EHT force field, both the proteins and small molecule structures were protonated. The MOE plugin ‘Dock’ is used to study the interactions between proteins and molecules. The induced fitting docking protocol was used for molecular docking, with the triangle matching algorithm used to generate docking patterns. The London *δ* G scoring function was used to calculate the binding energy of each docking pose, and the top 5 docking poses are retained. Finally, the GBVI/LAS *δ* G scoring function was used to further optimize these docking postures under the induction fitting algorithm, in order to calculate the binding affinity of the optimized docking poses. Based on the defined docking region, compound CZ-2401 selects the most similar conformation as the output result.

### 2.19 Microscale thermophoresis (MST)

MST was utilized to determine the equilibrium constant for the interaction between the molecule and protein. HNF4*α* and HNF4*α* mutations (K342A/W349A/E353A) (HY-P701139A; HY-P7S0852, MedChemExpress, Monmouth Junction, USA) were labeled using the Protein Labeling Kit RED-NHS. CZ-2401 was serially diluted in a 50 mmol/L HEPES buffer (pH 7.4, containing 0.05% Tween 20) and mixed with an equal volume of HNF4*α* protein, followed by incubation at room temperature for 20 min. The resulting mixture was then loaded into capillaries (Monolith NT.115 Capillary, NanoTemper Technologies, Munich, Germany). Thermophoresis signals were recorded, and the dissociation constant (*K*_D_) was calculated using a Nano Temper Monolith NT.115 instrument (NanoTemper Technologies, Munich, Germany), in accordance with the manufacturer’s instructions.

### 2.20 Statistical analyses

All statistical analyses were performed using GraphPad Prism 9.0 (GraphPad Software, San Diego, USA). Data represent at least three biologically independent experiments and are expressed as mean ± standard deviation (SD) or mean ± standard error of mean (SEM). Group comparisons were analyzed by an unpaired two-tailed Student’s *t*-test. Tumor growth dynamics were assessed using two-way ANOVA. TRIM47 expression levels in hepatocellular carcinoma (LIHC) were evaluated by the Wilcoxon rank-sum test. Correlation analyses utilized a two-tailed Spearman’s rank correlation test. Survival outcomes were determined *via* Kaplan–Meier methodology with log-rank testing. Specific statistical approaches and sample sizes (*n*) are detailed in figure legends. Statistical significance was defined as *P* < 0.05 and is explicitly annotated in figures.

## 3 Results

### 3.1 TRIM47 interacts with HNF4α and negatively regulates its function in HCC cells

**Figure 1:**
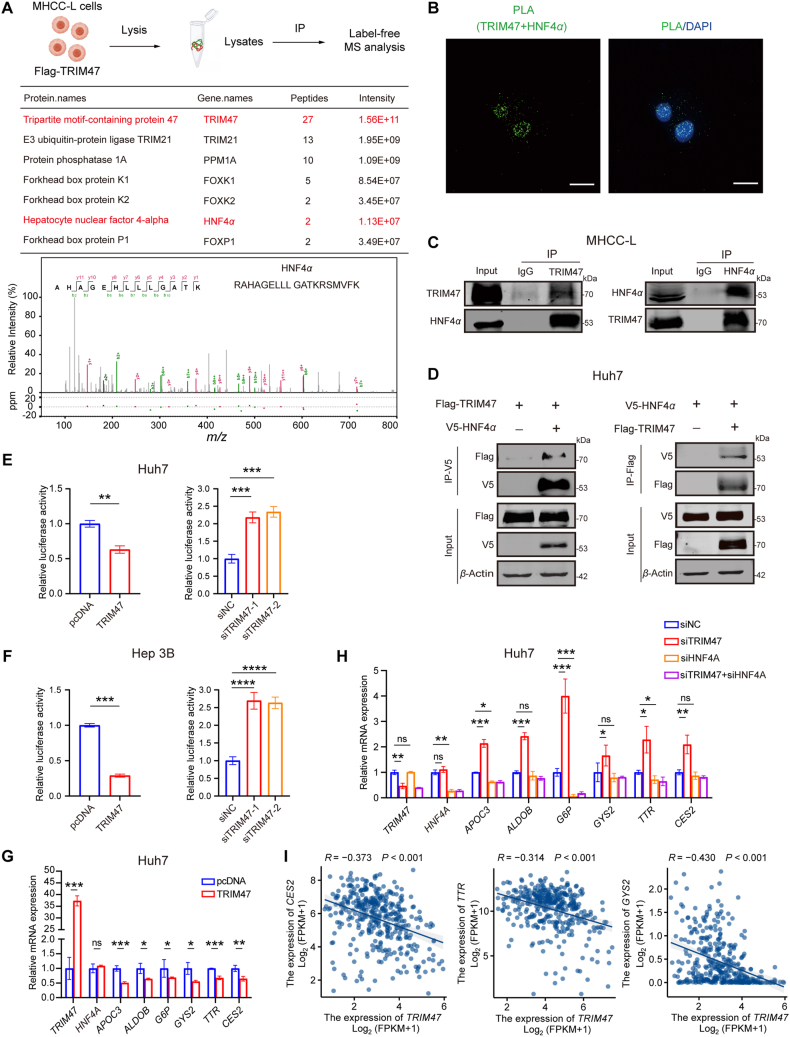
TRIM47 interacts with HNF4*α* and negatively regulates its function in HCC cells. (A) Schematic diagram of the experimental design for IP-MS analysis. Potential TRIM47-interacting proteins identified by MS analysis (top). Mass spectrometry analysis of an HNF4*α* peptide in Flag-TRIM47 precipitate (bottom). (B) *In situ* PLA signals of transfected V5-HNF4*α* and Flag-TRIM47 in Huh7 cells. Scale bar, 50 μm. (C) Co-immunoprecipitation of HNF4*α* and TRIM47 in MHCC-L cells. (D) Co-immunoprecipitation of transfected Flag-TRIM47 and V5-HNF4*α* in Huh7 cells. (E, F) Relative luciferase reporter activity of HNF4*α* in Huh7 cells (E) or Hep3B cells (F) transfected with TRIM47 or siTRIM47 for 48 h. (G, H) Relative mRNA levels of *HNF4A* and hepatocyte markers in Huh7 cells transfected with TRIM47 (G) siTRIM47, or/and siHNF4*α* (H) for 48h. (I) Gene correlation analysis between *TRIM47* and *HNF4A* target genes, including *CES2*, *TTR,* and *GYS2*, was performed using data from the TCGA-LIHC database. Statistical analyses were performed using two-sided unpaired Student’s *t*-test (E–G) and two-tailed Spearman’s correlation test (I). Data are presented as mean ± SD. *n* = 3 biologically independent samples (E–G), or 424 samples in LIHC (I). ∗*P* < 0.05, ∗∗*P* < 0.01, ∗∗∗*P* < 0.001, ∗∗∗∗*P* < 0.0001, ns indicates not significant.

When assessing the clinical significance of TRIM47 expression in HCC specimens, we observed that TRIM47 levels were significantly elevated compared to paracancerous tissues, and this upregulation was associated with shorter overall survival (OS) (Supporting Information Fig. S1), aligning with the recent literature^14^^,^^15^. Clinicopathological analysis further revealed that increased TRIM47 expression correlated significantly with aggressive clinical and pathological features, including reduced encapsulation (*P* = 0.034) and increased tumor recurrence (*P* < 0.001) (Supporting Information Table S4). Based on the protein level of TRIM47 in six HCC lines, we reinforced TRIM47 expression in Huh7 cells using lentivirus TRIM47 (LV-TRIM47), constructed TRIM47 knockout (TRIM47-KO) Huh7 cells *via* CRISPR-Cas9 technology and conducted TRIM47 knockdown in Hep3B cells, and then confirmed that TRIM47 promoted the malignant phenotypes of HCC cells *in vitro* (Supporting Information Figs. S2 and S3). Consistently, tumors in nude mice subcutaneously transplanted with TRIM47-KO-Huh7 cells were significantly smaller than those in the control group (Supporting Information Fig. S4). To further investigate the roles and underlying mechanisms of TRIM47 in HCC, we engineered MHCC-L cells to express Flag-tagged TRIM47 and performed immunoprecipitation and mass spectrometry analyses to identify its interacting partners. In addition to the previously reported partners TRIM21, PPM1A, and FOX family^11^^,^^33^^,^^34^, we were excited to discover the transcription factor HNF4*α* as a potential interacting partner of TRIM47 (Fig. 1A). Moreover, an *in situ* proximity ligation assay (PLA) in Huh7 cells using antibodies against TRIM47 and HNF4*α* revealed strong signals, indicating their physical interaction *in vivo* (Fig. 1B). Co-IP confirmed a strong interaction between TRIM47 and HNF4*α* in both MHCC-L cells and Flag-TRIM47/V5-HNF4*α*-transfected Huh7 cells (Fig. 1C and D).

As HNF4*α* is a key modulator in HCC and an attractive therapeutic target, we further investigated the role of TRIM47 in regulating HNF4*α* function, based on our identification of the interaction between TRIM47 and HNF4*α*. The luciferase reporter assay in Huh7 and Hep3B cells showed that TRIM47 overexpression significantly suppressed HNF4*α* transcriptional activity, while TRIM47 knockdown enhanced it (Fig. 1E and F). qRT-PCR analyses confirmed that overexpression of TRIM47 inhibited the expression of the reported *HNF4A* target genes in HCC cells (Fig. 1G). Moreover, TRIM47 knockdown upregulated *HNF4A*-target genes, while concurrent *HNF4A* depletion abolished these transcriptional changes (Fig. 1H). Consistently, data from TCGA and qRT-PCR analysis of 90 HCC tissues showed a negative correlation between *TRIM47* expression and the mRNA levels of *HNF4A* target genes, including carboxylesterase 2 (*CES2*), transthyretin (*TTR*), and glycogen synthase 2 (*GYS2*) in HCC (Fig. 1I and Supporting Information Fig. S5), confirming the suppressive role of TRIM47 on HNF4*α* function.

### 3.2 TRIM47 suppresses the protein levels of HNF4α in HCC

**Figure 2:**
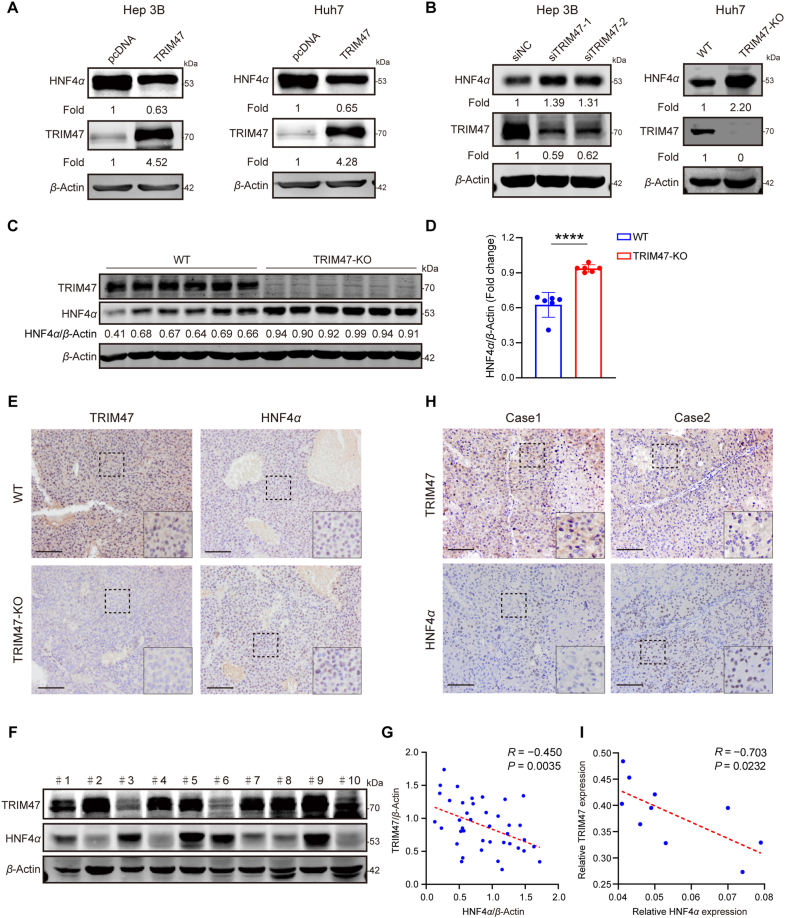
TRIM47 suppresses the protein levels of HNF4*α* in HCC. (A) Western blotting analysis of HNF4*α* expression in Hep3B and Huh7 cells transfected with TRIM47 or pcDNA3.0 plasmids for 72 h. The HNF4*α* expression was normalized with *β*-actin, and the fold of pcDNA-treated cells was adjusted to 1. (B) Western blotting analysis of HNF4*α* expression in Hep3B cells transfected with siTRIM47 or siNC (left) for 72 h. HNF4*α* protein levels in wild-type (WT) and TRIM47-knockout (TRIM47-KO) Huh7 cells (right). The HNF4*α* expression was normalized with *β*-Actin, and the vehicle control was adjusted to 1. (C) Representative Western blotting of TRIM47 and HNF4*α* in Huh7 xenograft tissues from mice in the indicated groups (*n* = 6). (D) Semi-quantification of HNF4*α* protein levels. (E) Representative images of IHC staining for TRIM47 and HNF4*α* in tumor xenografts. Scale bars, 100 μm. (F) Representative Western blotting analysis of HNF4*α* and TRIM47 protein levels in human HCC tissues. (G) The correlation of TRIM47 and HNF4*α* protein levels in HCC tissues by semi-quantitative analysis and Pearson correlation analysis (*R* = −0.450, *P* = 0.0035, *n* = 40). (H) The expression of TRIM47 and HNF4*α* proteins in HCC tissues was determined by immunohistochemistry. Scale bar, 100 μm. (I) The correlation of TRIM47 and HNF4*α* protein levels in HCC tissues was analyzed using Pearson correlation based on immunohistochemistry staining results (*R* = −0.703, *P* = 0.0232, *n* = 10). Statistical analyses were performed using two-sided unpaired Student’s *t*-test (D), two-tailed Spearman’s correlation test (G, I). Data are presented as mean ± SEM. *n* = 6 biologically independent samples (D), 40 (G), and 10 (I) biologically independent samples, ∗∗∗∗*P* < 0.0001.

To investigate the downstream effects of the TRIM47–HNF4*α* interaction, we measured the expression of HNF4*α* in HCC cells with TRIM47 overexpression, knockdown, or knockout. Results showed that TRIM47 overexpression significantly decreased (Fig. 2A), while TRIM47 knockdown and knockout increased the protein levels of HNF4*α* in HCC cells (Fig. 2B). Significantly, the modulation of TRIM47 levels did not induce any observable alterations in the expression of *HNF4A* mRNA (Fig. 1G and H, Supporting Information Fig. S6A). We also observed that the levels of HNF4*α* were upregulated in tumors of nude mice subcutaneously transplanted with TRIM47 KO-Huh7 cells compared to those of the control group (Fig. 2C–E). Furthermore, western blotting analysis of 40 cases of HCC tissues, along with IHC analysis of 10 HCC samples, demonstrated an inverse relationship between TRIM47 and HNF4*α* protein levels in human HCC tissues (Fig. 2F–I and Fig. S6B). These findings imply that TRIM47 may facilitate HCC progression by reducing the protein of HNF4*α in vivo*.

### 3.3 TRIM47 mediates the K48-linked ubiquitination and proteasomal degradation of HNF4α

**Figure 3:**
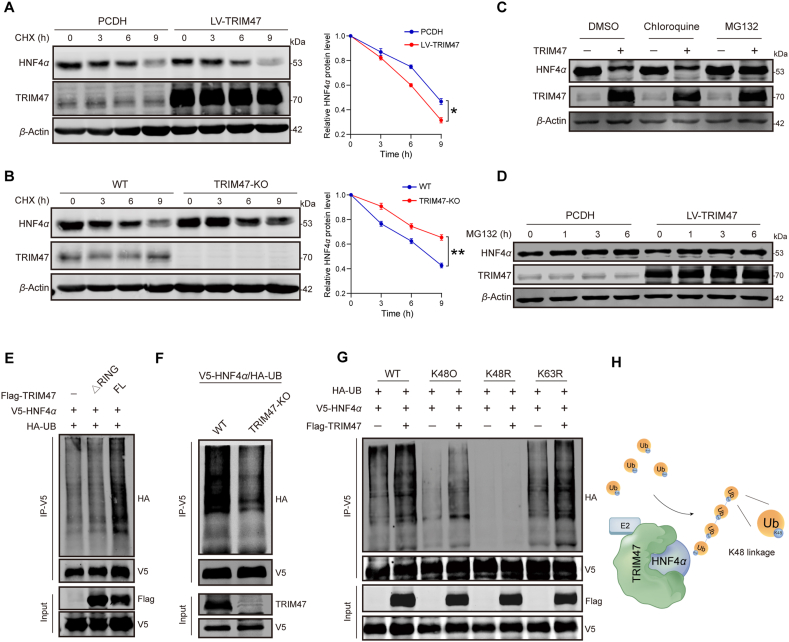
TRIM47 mediates the K48-linked ubiquitination and proteasomal degradation of HNF4*α*. (A) Huh7 cells were infected with control lentivirus (PCDH) or lentivirus expressing TRIM47 (LV-TRIM47) and subsequently treated with cycloheximide (CHX, 20 μg/mL) for 0, 3, 6, and 9 h. Western blotting analysis was performed to assess HNF4*α* protein levels (left). Semi-quantitative analysis of HNF4*α* protein levels after TRIM47 overexpression based on the CHX treatment assays (right). (B) Huh7 cells and TRIM47-KO Huh7 cells were treated with CHX (20 μg/mL) for 0, 3, 6, and 9 h. Western blotting was performed to evaluate HNF4*α* protein levels (left). Semi-quantitative analysis of HNF4*α* protein levels after TRIM47 knockout based on the CHX-treated assay (right). (C) Representative Western blotting analysis of HNF4*α* protein levels in Huh7 cells transfected with TRIM47 plasmids and treated with DMSO, chloroquine (20 μmol/L), or MG132 (20 μmol/L) for 6 h. (D) Representative Western blotting of HNF4*α* and TRIM47 in Huh7 cells infected with PCDH or LV-TRIM47 and treated with MG132 (20 μmol/L) for the indicated time. (E) HEK293T cells were co-transfected with V5-HNF4*α*, HA-UB, and Flag-TRIM47 ΔRing (Δ1–85) mutant or Flag-TRIM47 for 48 h. Cell lysates were used for immunoprecipitation with V5 antibody and Western blotting with the indicated antibodies. (F) Ubiquitination of HNF4*α* was assessed in WT or TRIM47-KO Huh7 cells transfected with V5-HNF4*α* and HA-UB plasmids for 48 h. (G) Ubiquitination of HNF4*α* in HEK293T cells co-transfected with TRIM47, V5-HNF4*α,* and plasmids expressing WT HA-Ub or HA-Ub with one of the following mutations: K48O, K48R, or K63R. The ubiquitin levels were detected at 48 h post-transfection. (H) Schematic diagram of the mechanism by which TRIM47 catalyzes HNF4*α* ubiquitination. Statistical analyses were performed using a two-sided unpaired Student’s *t*-test (A, B). The data presented in (A, B) are representative of three independent experiments and are presented as mean ± SD. ∗*P* < 0.05, ∗∗*P* < 0.01.

Given the interaction between TRIM47 and HNF4*α*, we hypothesized that TRIM47, as an E3 ligase, decreases HNF4*α* protein levels *via* ubiquitin-proteasome degradation. Cycloheximide chase assays showed that TRIM47 overexpression significantly accelerated HNF4*α* protein degradation (Fig. 3A). Conversely, the degradation of HNF4*α* was markedly slowed down when TRIM47 was knockout in Huh7 cells (Fig. 3B). Furthermore, treating TRIM47-overexpressing Huh7 cells with the proteasome inhibitor MG132 resulted in a significant rebound of HNF4*α* protein levels, whereas the lysosome inhibitor Chloroquine had no effect, confirming that TRIM47 induces HNF4*α* degradation *via* the proteasome (Fig. 3C and D). Ubiquitination assays further showed that TRIM47 overexpression facilitated ubiquitination of HNF4*α* in hepatocytes, while TRIM47 knockout reduced it (Fig. 3E and F). As the Ring domain of TRIM47 is essential for E2 enzyme recruitment and ubiquitin transfer^35^, a Ring domain-deleted TRIM47 mutant (ΔRING) was constructed to further validate the effect of TRIM47 on HNF4*α* ubiquitination. The data showed that the ΔRING mutation abolished TRIM47-induced ubiquitination of HNF4*α* (Fig. 3E), confirming that TRIM47 is an E3 ubiquitin ligase of HNF4*α*. Lys48-linked polyubiquitin is a canonical modification that targets substrates for proteasome degradation^36^. Screening ubiquitin mutants for lysine ubiquitination demonstrated that the K48R mutation of ubiquitin completely abolished TRIM47-mediated ubiquitination of HNF4*α* in V5-HNF4*α*/Flag-TRIM47-overexpressing HEK293T cells (Fig. 3G and H). Collectively, these data suggested that TRIM47 mainly promotes K48-linked ubiquitination of HNF4*α*.

### 3.4 Blocking the ubiquitination of HNF4α alleviates the detrimental effects of TRIM47 on HCC

**Figure 4:**
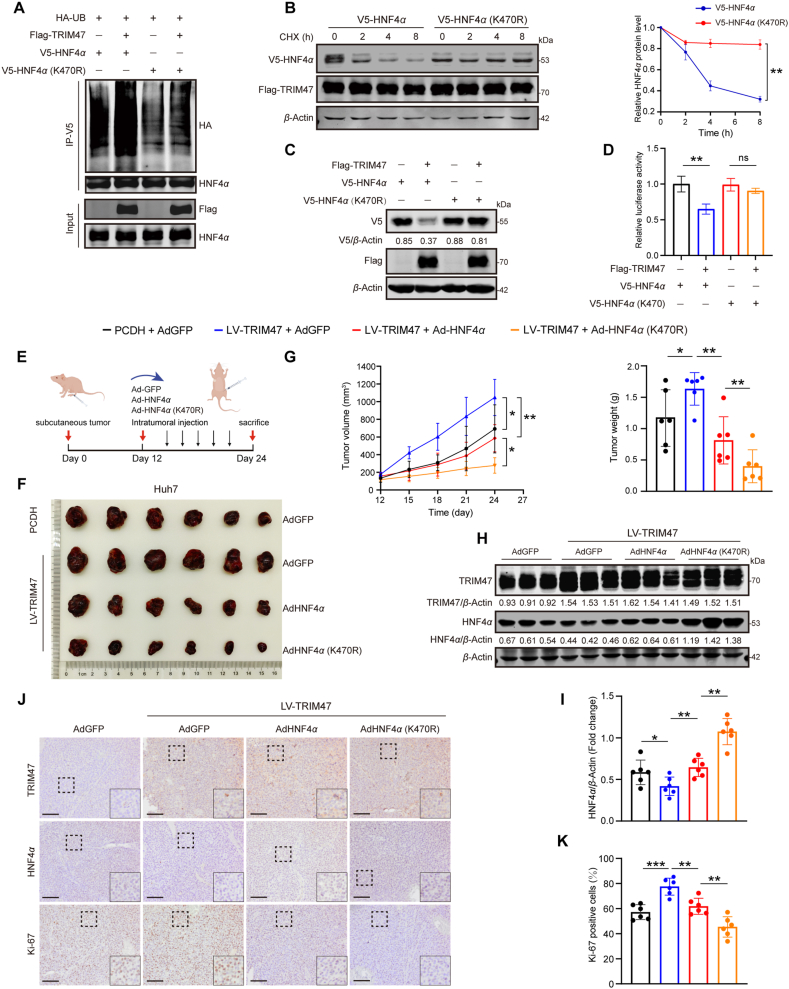
Blocking the ubiquitination of HNF4*α* alleviates the detrimental effects of TRIM47 on HCC. (A) Ubiquitination assays were conducted to evaluate the ubiquitination levels of HNF4*α* in Huh7 cells transfected with V5-HNF4*α* or V5-HNF4*α*-K470R for 48 h, in the presence or absence of TRIM47 overexpression. (B)The protein levels of HNF4*α* were examined in TRIM47-overexpressing HEK293T cells transfected with V5-HNF4*α* or V5-HNF4*α*-K470R plasmids and treated with CHX (20 μg/mL) for 0, 2, 4, and 8 h (left). The semi-quantitative analysis of HNF4*α* protein levels following TRIM47 overexpression in the CHX-treated assay (right). (C) Western blotting analysis of HNF4*α* protein levels in Huh7 cells transfected with HNF4*α* or HNF4*α*-K470R together with Flag-TRIM47 or control plasmids for 72 h. (D) Luciferase reporter assays were conducted to assess *HNF4A* promoter activity in Huh7 cells transfected with the indicated plasmids for 48 h. (E, F) Images of xenograft tumors from mice inoculated with Huh7 cells infected with control lentivirus (PCDH) or lentivirus TRIM47 (LV-TRIM47), followed by intratumoral injection of adenovirus expressing GFP, HNF4*α*, or HNF4*α*-K470R. (G) Tumor volume was measured at specified time points after subcutaneous implantation and intratumoral injection (left). Tumor weight was assessed at the time of sacrifice (right). (H) Representative Western blotting analysis of HNF4*α* and TRIM47 protein levels in xenograft tumors. (I) Semi-quantitative analysis of HNF4*α* protein levels in the indicated groups of xenograft tumors. (J) The expression of HNF4*α*, TRIM47, and Ki-67 proteins in xenograft tumors was assessed by immunohistochemistry. Scale bar, 100 μm. (K) Quantitative analysis of the areas of Ki-67-positive cells in transplanted tumors. Statistical analyses were performed using a two-tailed Student’s *t*-test (B, D, G right, I, K) or two-way ANOVA with multiple comparisons (G left). The data presented in B are representative of three independent experiments and are presented as mean ± SD for (B, D) and mean ± SEM for (G, I, K). ∗*P* < 0.05, ∗∗*P* < 0.01, ∗∗∗*P* < 0.001; ns, not significant.

Our recent study revealed that Lys470 serves as a critical ubiquitination site on HNF4*α* during the progression of NAFLD^25^. Here, we investigated whether TRIM47 also mediates the ubiquitination of HNF4*α* at this site using the plasmid containing an HNF4*α* lysine-to-arginine mutation at position 470 (K470R). As expected, the *in vitro* ubiquitin assay revealed that the K470R mutation significantly reduced TRIM47-mediated ubiquitination of HNF4*α* (Fig. 4A). Moreover, the K470R mutation significantly delayed HNF4*α* degradation in CHX-treated Huh7 cells overexpressing TRIM47 (Fig. 4B), leading to the restoration of HNF4*α* protein levels (Fig. 4C). Consistently, luciferase reporter assays showed that the K470R mutant significantly reinstated TRIM47-mediated inhibition of HNF4*α* transcriptional activity (Fig. 4D).

Given that TRIM47 mediates the degradation of HNF4*α*, we aimed to investigate whether inhibiting this degradation could attenuate TRIM47-driven HCC progression. Following the infection of TRIM47-overexpressing Huh7 cells with either GFP, HNF4*α*-WT (AdHNF4*α*), or HNF4*α*-K470R (AdHNF4*α*-K470R) adenoviruses, we found that the promoting effects of TRIM47 on proliferation, migration, invasion, and colony formation were abolished by the HNF4*α*-K470R mutant (Supporting Information Fig. S7A–S7F). We next evaluated whether blocking *HNF4α* ubiquitination could reverse the oncogenic effects of TRIM47 *in vivo*. Nude mice were subcutaneously transplanted with Huh7 cells infected with LV-TRIM47 or PCDH, followed by intratumoral injections of Ad-GFP, Ad-HNF4*α,* or AdHNF4*α*-K470R (Fig. 4E). Consistent with the *in vitro* results, AdHNF4*α*-K470R reversed the TRIM47-induced increase in tumor growth, volume, and weight (Fig. 4F and G). Notably, Western blotting and IHC analysis of tumors revealed that HNF4*α* protein levels decreased by TRIM47 overexpression but were restored by AdHNF4*α*-K470R (Fig. 4H–J), while mRNA levels showed no significant change (Supporting Information Fig. S7G). IHC staining of Ki-67 further supported that AdHNF4*α*-K470R strongly inhibited the TRIM47-induced tumor proliferation in mice (Fig. 4J and K). Taken together, these data demonstrate that blocking HNF4*α* ubiquitination by mutating K470 effectively stabilizes HNF4*α*, thereby preventing the progression of TRIM47-aggravated HCC.

### 3.5 The mode of HNF4α–TRIM47 interaction

**Figure 5:**
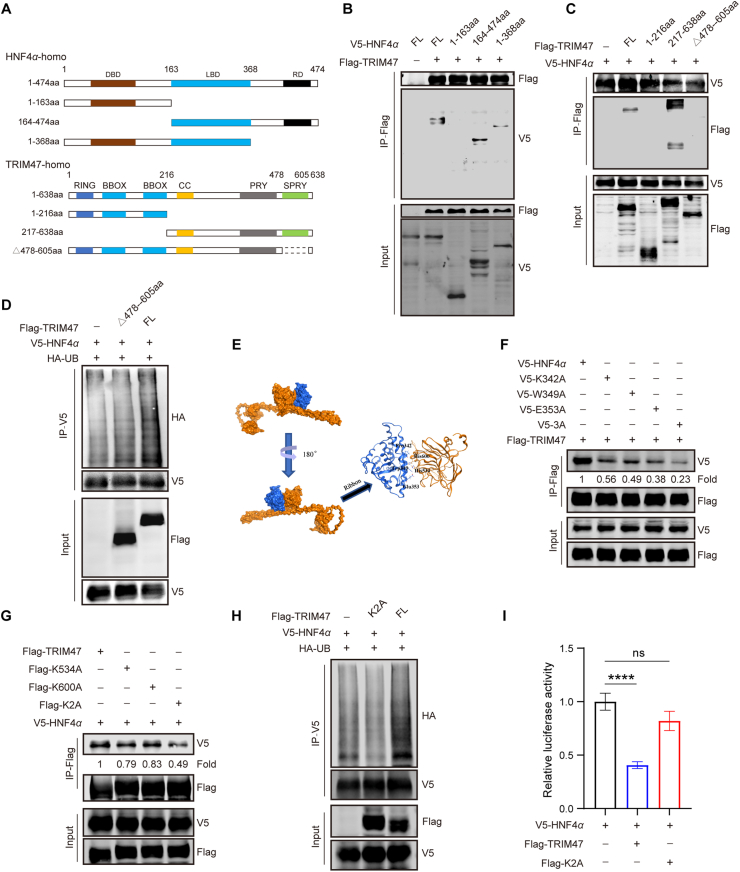
The model of HNF4*α*–TRIM47 interaction. (A) Mapping of TRIM47 regions interacting with HNF4*α*. Schematic diagrams of full-length HNF4*α* and its deletion mutants (top), and full-length TRIM47 and its deletion mutants (bottom). (B) HEK293T cells were co-transfected with Flag-TRIM47 and V5-HNF4*α* or truncated HNF4*α* for 48 h. Cell lysates were used for immunoprecipitation with anti-Flag agarose affinity gels and Western blotting with the indicated antibodies. (C) HEK293T cells were co-transfected with V5-HNF4*α* and Flag-TRIM47 or truncated TRIM47 for 48 h, and subjected to immunoprecipitation with anti-V5 agarose affinity gels and Western blotting with the indicated antibodies. (D) Ubiquitination levels of HNF4*α* in HEK293T cells co-transfected withV5-HNF4*α*, along with full-length Flag-TRIM47 or the Flag-TRIM47 Δ478–605 mutant for 48 h. (E) Docking model predicting the residues involved in the binding between TRIM47 and HNF4*α*. (F) Interactions between TRIM47 and HNF4*α* in HEK293T cells transfected with V5*-*HNF4*α* or its mutants (V5-K300A, V5-W349A, V5-E353A, and V5-3A) and Flag-TRIM47 for 48h were detected using Co-IP and Western blotting analysis. Cell lysates were used for immunoprecipitation with anti-Flag or anti-V5 agarose affinity gels, followed by Western blotting with the indicated antibodies. (G) Co-IP of transfected Flag-TRIM47 or TRIM47 mutants (Flag-K534A, Flag-K600A, and Flag-K2A) and HNF4*α* in HEK293T for 48 h. K2A indicates K534A and K600A double mutants. (H) Ubiquitination levels of HNF4*α* in HEK293T cells overexpressing full-length Flag-TRIM47 or Flag-K2A mutant for 48 h. (I) Relative luciferase reporter activity of HNF4*α* in HEK293T cells transfected with V5*-*HNF4*α* and Flag-TRIM47 or the Flag-K2A mutant for 48 h. Statistical analyses were performed using a two-tailed Student’s *t*-test (I). Data are presented as mean ± SD for I. ∗∗∗∗*P* < 0.0001; ns, not significant.

Having established that TRIM47 drives HCC progression by binding HNF4*α* and promoting its ubiquitination-dependent degradation, we posited that pharmacological inhibitors targeting the TRIM47–HNF4*α* interaction might offer therapeutic potential. To elucidate the structural basis of this interaction, we engineered truncated HNF4*α* and TRIM47 plasmids based on domain schematics (Fig. 5A). Through the expression of various V5-HNF4*α* truncation mutants and subsequent Co-IP analysis, we pinpointed amino acids 163-368 of HNF4*α*, comprising the ligand-binding domain, interacted with TRIM47 (Fig. 5B). Correspondingly, we revealed that amino acids 478–605, which form the SPRY domain of TRIM47, as crucial for its interaction with HNF4*α* (Fig. 5C), and found that deletion of this domain completely abolished HNF4*α* ubiquitination (Fig. 5D). Subsequent molecular docking studies revealed that the HNF4*α*–TRIM47 interaction involves K342, W349 and E353 in HNF4*α*, along with K534 and K600 in TRIM47 (Fig. 5E). Actually, the overexpression of the V5-tagged HNF4*α*-K342A/W349A/E353A mutant (HNF4*α*-3A) and the introduction of double mutations K534A and K600A in TRIM47 (TRIM47-K2A) both resulted in a marked reduction of the interaction between HNF4*α* and TRIM47 (Fig. 5F and G). Moreover, TRIM47-K2A inhibited the TRIM47-mediated ubiquitination of HNF4*α*, leading to the restoration of HNF4*α* transcriptional activity (Fig. 5H and I). Overall, our data clarified the binding sites involved in the HNF4*α*–TRIM47 interaction.

### 3.6 CZ-2401 enhances HNF4α stability by blocking HNF4α–TRIM47 interaction

**Figure 6:**
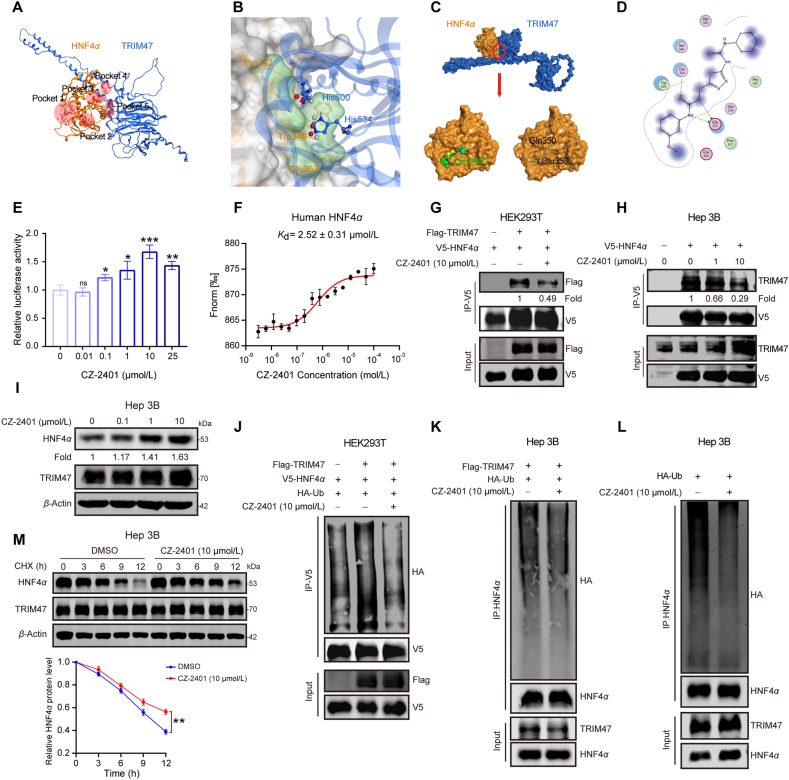
CZ-2401 enhances HNF4*α* stability by blocking HNF4*α*–TRIM47 interaction. (A) The top five predicted binding pockets for potential inhibitors were identified through structural modeling (MOE-Site Finder software). (B) Detailed visualization of Binding Pocket 5. (C) Docking model predicting the residues involved in the binding of CZ-2401 to the TRIM47–HNF4*α* complex. (D) Structural diagram of the small molecule compound CZ-2401. (E) The relative luciferase reporter activity of HNF4*α* in Hep3B cells treated with different concentrations of CZ-2401 for 24 h. (F) MST assay detecting the binding of CZ-2401 to HNF4*α in vitro*. (G) Co-IP of Flag-TRIM47 and V5-HNF4*α* in HEK293T cells treated with CZ-2401 (10 μmol/L) for 24 h. (H) Co-IP of V5-HNF4*α* and endogenous TRIM47 in Hep3B cells treated with the indicated concentrations of CZ-2401 for 24 h. (I) Western blotting analysis of HNF4*α* in Hep3B cells treated with different concentrations of CZ-2401 for 24 h. (J) Ubiquitination of HNF4*α* in HEK293T cells transfected with the indicated plasmids after treatment with CZ-2401 for 24h. (K, L) Ubiquitination of HNF4*α* in Hep3B cells transfected with the indicated plasmids after treatment with CZ-2401 for 24 h. (M) Hep3B cells were treated with 10 μmol/L CZ-2401 for 24 h, followed by cycloheximide (CHX, 20 μg/mL) treatment at time points of 0, 3, 6, 9, and 12 h. Western blotting analysis was performed to evaluate HNF4*α* protein levels (left). Semi-quantitative assessment of HNF4*α* protein was conducted based on the CHX-treated assay (right). The relative values were calculated with the vehicle control adjusted to 1. Statistical analyses were performed using a two-tailed Student’s *t*-test (E, M). The data presented in M are representative of three independent experiments and are presented as mean ± SD for (E, M). ∗*P* < 0.05, ∗∗*P* < 0.01, ∗∗∗*P* < 0.001; ns, not significant.

Subsequently, we focused on developing a small-molecule inhibitor targeting the HNF4*α*–TRIM47 interaction by analyzing the binding mode with MOE-Site Finder software. This analysis identified a potential inhibitor binding pocket on HNF4*α* (Pocket 5), encompassing the core amino acids at the binding interface within the HNF4*α*–TRIM47 complex (Fig. 6A and B). Then, we performed virtual screening (VS) of a chemical library comprising 29,500 small molecules to identify compounds targeting the Pocket 5 of HNF4*α*. This screen initially yielded 20 potential candidates, which were subsequently evaluated using luciferase reporter assays and western blotting to assess their effects on HNF4*α* activity (Supporting Information Fig. S8A and S8B). Among these, CZ-2401 emerged as the lead candidate due to its dose-dependent enhancement of HNF4*α* transcriptional activity and HNF4*α* protein accumulation (Fig. 6C–E). A microscale thermophoresis (MST) assay further confirms that CZ-2401 exhibits a strong binding affinity for HNF4*α* (*K*_d_ = 2.52 ± 0.31 μmol/L) (Fig. 6F), while mutagenesis of key binding residues of HNF4*α* Pocket 5 (K342A/W349A/E353A) drastically reduced this interaction (*K*_d_ = 8.33 ± 0.69 μmol/L) (Fig. S8C). Consistently, luciferase reporter assays demonstrated that the HNF4*α*-3A mutation significantly attenuated the CZ-2401-induced activation of HNF4*α* transcriptional activity (Fig. S8D). The interaction between TRIM47 and HNF4*α* was significantly inhibited by CZ-2401, resulting in the accumulation of HNF4*α* protein (Fig. 6G–I and Fig. S8E). Further ubiquitination and CHX analysis revealed that CZ-2401 effectively diminished TRIM47-mediated ubiquitination modification of HNF4*α* (Fig. 6J–L), thereby slowing down the degradation rate of HNF4*α* (Fig. 6M). Collectively, CZ-2401 stabilizes HNF4*α* protein by blocking the binding of HNF4*α* with TRIM47 *in vitro*.

### 3.7 CZ-2401 treatment ameliorates the TRIM47-driven HCC progression

**Figure 7:**
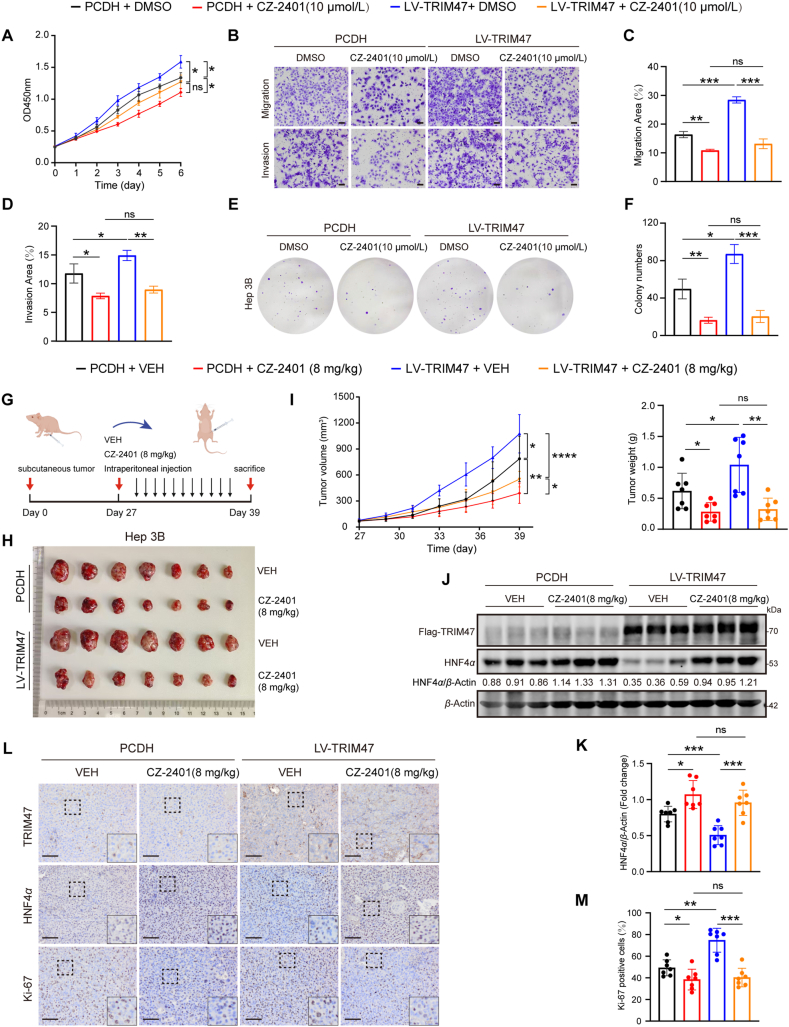
CZ-2401 treatment ameliorates the TRIM47-driven HCC progression. (A) CCK-8 assays were conducted on Hep3B cells infected with PCDH or LV-TRIM47, followed by treatment with DMSO or CZ-2401 at 10 μmol/L. (B–D) Migration (top) and invasion (bottom) assays of Hep3B cells infected with PCDH or LV-TRIM47, followed by treatment with DMSO or CZ-2401 (10 μmol/L) for 72 h. Scale bar, 100 μm. Statistical results of migration (C) or invasion (D). (E, F) Representative images (E) of colony formation assays and statistical results (F) for Hep3B cells infected with PCDH or LV-TRIM47, cells were treated with DMSO or CZ-2401 (10 μmol/L) for 14 days, with medium replenished every other day to maintain drug concentrations. (G, H) Images of xenograft tumors from mice inoculated with Hep3B cells infected with PCDH or LV-TRIM47, with each mouse receiving a daily intraperitoneal injection of CZ-2401 (8 mg/kg) or saline solution for 12 days. (I) Tumor volume was measured at the indicated time points following subcutaneous implantation and intraperitoneal injection (left). Tumor weight was assessed at the time of sacrifice (right). (J, K) Representative Western blotting analysis of HNF4*α* and TRIM47 protein levels in xenograft tumors (J) and semi-quantitative analysis of HNF4*α* protein levels in the indicated groups (K). (L) The expression levels of HNF4*α*, TRIM47, and Ki-67 proteins in xenograft tumors were evaluated using immunohistochemistry. Scale bar, 100 μm. (M) Quantitative analysis of the areas of Ki-67-positive cells in transplanted tumors. Statistical analyses were performed using two-way ANOVA with multiple comparisons (A, I left), two-tailed Student’s *t*-test (C, D, F, I right, K, M). Data are presented as mean ± SD for (A, C, D, F) and mean ± SEM for (I, K, M). ∗*P* < 0.05, ∗∗*P* < 0.01, ∗∗∗*P* < 0.001; ns, not significant.

The marked stabilization of HNF4*α* protein by the HNF4*α*–TRIM47 interaction inhibitor CZ-2401 prompted us to explore its potential therapeutic benefits for HCC. The cytotoxicity assessments revealed that CZ-2401 exhibited potent cytotoxic effects in both Huh7 and Hep3B cells but a minimal cytotoxicity in HEK293T cells with absent HNF4*α* expression (Supporting Information Fig. S9A). In addition, HNF4*α* knockdown in Huh7 and Hep3B cells substantially attenuated CZ-2401-induced cytotoxicity, confirming that CZ-2401 specifically targets HNF4*α* (Fig. S9B). Then, we assessed the effects of CZ-2401 treatment on Hep3B cells overexpressing TRIM47 *in vitro*. Compared to the control group, CZ-2401 treatment significantly attenuated the stimulatory effects of TRIM47 on cell proliferation, migration, invasion, and colony formation (Fig. 7A–F). Flow cytometry further confirmed that CZ-2401 suppressed proliferation and induced apoptosis of HCC cells, reinforcing its anti-tumorigenic efficacy (Fig. S9C–S9E). We next established a mouse xenograft model by subcutaneously inoculating nude mice with Hep3B cells, either with or without TRIM47 overexpression, followed by daily intraperitoneal injections of saline solution (VEH) or CZ-2401 (8 mg/kg) (Fig. 7G). As expected, while TRIM47 overexpression exacerbated tumor growth in xenografts, as evidenced by increases in tumor size, volume, and weight, this effect was significantly diminished by CZ-2401 treatment (Fig. 7H and I). We also assessed the expression of HNF4*α* in the xenograft tumors and noted a significant reduction in HNF4*α* protein levels in the TRIM47-overexpressing (LV-TRIM47) group compared to controls (PCDH). Conversely, CZ-2401 treatment led to a significant increase in HNF4*α* levels, even in the presence of TRIM47 overexpression (Fig. 7J and K). IHC for Ki-67 staining further confirmed the inhibitory effect of CZ-2401 on the TRIM47-accelerated proliferation of tumor cells (Fig. 7L and M). Taken together, these data demonstrate that CZ-2401, a selective inhibitor targeting the HNF4*α*–TRIM47 interaction, displays the potential for HCC therapy.

## 4 Discussion

In recent years, for individuals diagnosed with unresectable hepatocellular carcinoma, systemic anti-HCC therapy has emerged as the standard treatment^37^. Although multikinase inhibitors such as sorafenib and regorafenib have been approved for the first-line and second-line treatment of advanced-stage HCC, the majority of responders ultimately develop resistance to these therapies^1^^,^^38^. Currently, the emergence of novel therapeutic targets for HCC has attracted considerable attention from the scientific community, underscoring the continuous advancements in liver cancer research^39^. Herein, we demonstrated that TRIM47 promotes HCC progression by binding HNF4*α* and facilitating its ubiquitination, underscoring the potential of blocking HNF4*α* degradation as a therapeutic strategy for HCC.

In hepatocellular carcinoma cells, many TRIM proteins are aberrantly expressed in tumors and regulate tumorigenesis and progression^40^. For instance, TRIM25, TRIM65, and TRIM8 have been shown to be elevated in the tissues of liver cancer patients and are correlated with poor prognosis^41^, ^42^, ^43^. In detail, TRIM25 facilitates the degradation of FBXW7*α* through mediating its K48 ubiquitination, thereby inhibiting FBXW7*α*’s role in Myc degradation and promoting the proliferation of HCC cells^41^. TRIM65 mediates the ubiquitination at the K44 site of NF2, accelerating NF2 degradation, inhibiting the phosphorylation of Yes-associated protein 1, and promoting the HCC progression^42^. Moreover, our recent study revealed that TRIM8 facilitates the malignant progression of HCC *via* K48-linked ubiquitination of HNF1*α*^43^. Notably, a study combining the TRIM family risk score with clinical parameters identified TRIM47 as a key mediator in the malignant progression of liver cancer^32^. Consistently, recent work by Cheng et al.^14^ showed that TRIM47, acting as a key regulator of ferroptosis sensitivity, promotes HCC progression by mediating the ubiquitination-mediated degradation of CDO1. In addition, a recent work showed that TRIM47 stabilizes the EMT-inducing transcription factor SNAI1 through CARM1-mediated methylation, thereby promoting tumor metastasis^15^. While these findings establish TRIM47 as a promising molecular target, the current studies remain limited by insufficient clinical validation, highlighting the need for translational research to bridge this gap between mechanistic discovery and therapeutic application. Here, we have also confirmed that elevated TRIM47 levels in HCC tissues are strongly correlated with malignant phenotypes. Significantly, we identify HNF4*α* as a novel ubiquitination substrate of TRIM47, revealing an alternative oncogenic pathway mediated by TRIM47 and further advancing our understanding of the molecular mechanisms underlying TRIM47-driven HCC progression.

HNF4*α* is a key transcription factor predominantly expressed in the liver, kidney, pancreas, and intestine, with its highest abundance found in hepatocytes^44^. It is considered the master regulator of hepatocyte proliferation, differentiation, and maturation, coordinating a multitude of hepatic transcriptomes and various extracellular and intracellular signaling pathways^45^. HNF4*α* functionality diminishes with the progression of liver disease, transitioning from steatosis to non-alcoholic steatohepatitis (NASH), culminating in cirrhosis and hepatocellular carcinoma^46^. Our previous studies demonstrated that *in vitro* adenovirus-mediated HNF4*α* overexpression in HCC cells reduced cell survival by inducing apoptosis, while *in vivo*, *HNF4A* gene delivery protected mice from liver metastasis and significantly inhibited tumor growth in a transplant model^20^. Notably, recent research has demonstrated that the collaborative function of HNF4*α*, HNF1*α*, and FOXA3 is pivotal in reprogramming HCC cells toward a hepatoid phenotype^47^. Current studies reported that HNF4*α* activity is regulated by multiple PTMs, including acetylation, phosphorylation, and arginine methylation^21^^,^^48^, ^49^, ^50^, ^51^. For instance, K458 acetylation^21^, phosphorylation at S78 mediated by PKC^48^, S304 mediated by AMPK^49^, or Y14 mediated by Src^50^ have been shown to suppress HNF4*α* activity. In contrast, R91 methylation by PRMT1 enhances its transcriptional activity^51^. Although the reduction of HNF4*α* is recognized as a critical event in the development of HCC, the intricate mechanisms underlying its downregulation, particularly concerning post-translational modifications, remain largely unexplored. Our recent research demonstrated that the Lys470 residue of HNF4*α* undergoes ubiquitination mediated by the TRIM8–TRIB3 E3 ligase complex, leading to HNF4*α* degradation during the progression of NAFLD^25^. Here, we propose a model for HNF4*α* degradation in HCC, wherein the same residue is also subject to K48-linked ubiquitination mediated by TRIM47. Furthermore, the HNF4*α*-K470R mutation abolishes the oncogenic role of TRIM47, highlighting the critical role of Lys470 ubiquitination in regulating HNF4*α* stability and providing a new starting point for developing HNF4*α* stabilizers. However, the HNF4*α*-K470R mutation may lack therapeutic properties in humans, as gene therapy is associated with difficulties in ensuring safe and targeted delivery, and potential immune response risks^52^. As an alternative, we propose the use of small-molecule compounds to stabilize HNF4*α*, thereby enhancing its potential for clinical translation.

Small molecule compounds, typically under 1000 Da, are playing an increasingly pivotal role in targeted cancer therapy due to their diverse mechanisms of action, broad applicability, and potential for continuous innovation^53^. Among these, the development of novel protein–protein interaction modulators in preclinical studies has become a noteworthy target^54^. For instance, Chen utilized molecular docking technology to discover novel KRAS/PDE*δ* inhibitors, which effectively disrupt the interaction between PDE*δ* and KRAS, thereby downregulating the phosphorylation levels of Akt and Erk and inducing cell apoptosis^55^. Herein, we have established a screening model based on HNF4*α* stability and identified the small molecule compound CZ-2401, which occupies the ligand-binding domain of HNF4*α*, as a potent inhibitor of the TRIM47–HNF4*α* interaction. Given the limited targeted approaches against the transcription factor HNF4*α*, our findings suggest that disrupting the TRIM47–HNF4*α* interaction may offer a novel therapeutic strategy for HCC and potentially other liver diseases by stabilizing HNF4*α*. However, the validation of tumor inhibition using patient-derived xenograft mouse models, along with systematic toxicological testing and pharmacokinetic studies of CZ-2401, is necessary for its future development as a therapeutic drug. It should be noted that CZ-2401 was screened based on the pocket of HNF4*α*, which interacts with TRIM47 to block the TRIM47–HNF4*α* interaction. It is possible that CZ-2401 may also inhibit the binding of HNF4*α* with other proteins, thereby targeting additional signaling pathways, which warrants further investigation. Further, since HNF4*α* plays a critical role in liver diseases such as NASH and liver cirrhosis, we will also explore the therapeutic effects of the CZ-2401 on these liver conditions to provide adequate experimental evidence for early clinical application.

## 5 Conclusions

Our study reveals that TRIM47 promotes hepatocellular carcinoma progression by targeting HNF4*α* for ubiquitin–proteasome degradation. Mutation of the HNF4*α* ubiquitination site at lysine 470 significantly impedes tumor growth. We also identified the SPRY domain of TRIM47 and the LBD domain of HNF4*α* as crucial for their interaction, providing insights into the binding site. Additionally, the small molecule compound CZ-2401, which inhibits the TRIM47–HNF4*α* interaction, effectively suppresses tumor progression both *in vitro* and *in vivo*, presenting CZ-2401 as a potential lead compound for future HCC therapeutics.

## Author contributions

Huanyu Hong: Investigation, Writing-original draft, Data curation, Formal analysis. Mengchao Xiao: Data curation, Formal analysis, Methodology. Hui Qian: Conceptualization, Validation. Siqi Tan: Investigation, Data curation. Sihan Wu: Investigation, Data curation. Fang Liu: Formal analysis. Xialu Hong: Methodology, Data curation. Shuqing Liu: Methodology, Data curation. Chenhong Ding: Methodology, Data curation. Keqi Wang: Conceptualization, Investigation, Writing-review & editing, Funding acquisition. Weifen Xie: Conceptualization, Resources, Supervision, Funding acquisition. Xin Zhang: Conceptualization, Resources, Supervision, Writing-review & editing, Funding acquisition.

## Conflicts of interest

The authors declare no conflicts of interest.

## Appendix A Supporting information

The following is the Supporting Information to this article:
