# Supplementary material for: Blocking TRIM47-mediated HNF4α degradation suppresses hepatocellular carcinoma progression
Source: Acta Pharm Sin B. 2025 Nov 1;16(2):913–29. doi: 10.1016/j.apsb.2025.10.045 (PMC12891897; doi:10.1016/j.apsb.2025.10.045)
Supplement: Multimedia component 1 [file mmc1.pdf]

Supporting Information for

Original article

Blocking TRIM47-mediated HNF4*α* degradation suppresses hepatocellular carcinoma progression

Huanyu Hong[a,†], Mengchao Xiao[b,†], Hui Qian[a], Siqi Tan[c], Sihan Wu[a], Fang Liu[a], Xialu Hong[a], Shuqing Liu[a], Chenhong Ding[b], Keqi Wang[a,*], Weifen Xie[a,*], Xin Zhang[a,*]

[a]*Department of Gastroenterology, Changzheng Hospital, Naval Medical University, Shanghai 200003, China*

[b]*Department of Gastroenterology, Shanghai East Hospital, School of Medicine, Tongji University, Shanghai 200092, China*

[c]*Department of Gastroenterology, Jiangnan University Medical Center (Wuxi No.2 People's Hospital), Wuxi, 214002, China*

Received 8 April 2025; received in revised form 12 May 2025; accepted 12 June 2025

*Corresponding authors.

E-mail addresses: zhang68@hotmail.com (Xin Zhang), weifenxie@medmail.com.cn (Weifen Xie), wangkeqi1001@163.com (Keqi Wang).

[†]These authors made equal contributions to this work.

1. Supplementary tables

Table S1 Primers used for qRT-PCR.

Gene(human)	Forward primer (5′–3′)	Reverse primer (5′–3′)
HNF4A	CTTCCTTCTTCATGCCAG	ACACGTCCCCATCTGAAG
TRIM47	AGAACGAGGTGATGGGGTT	CCTGGGAGGACTTGGTGAA
β-Actin	CATCCTGCGTCTGGACCT	GTACTTGCGCTCAGGAGGAG
ALDOB	ACAAGGTGCTGCGGGAATCA	ACTGGTGGGAGGGGTAGGTG
G-6-P	GGCTCCATGACTGTGGGATC	TTCAGCTGCACAGCCCAGAA
GYS2	CCAGTGGGAAGTCGAAGAAC	TTCTCTCCCCATTCATCTGC
TTR	TCAGAAAGGCTGCTGATGAC	AGTCGTTGGCTGTGAATACC
CES23	CTGGGGAGTCTTGTCCATGT	ATCCCTCACACCACTCCAAG
APOC3	GGGTACTCCTTGTTGTTGC	AAATCCCAGAACTCAGAGAAC

Table S2 Antibodies used for WB, IP, ICFC and IHC

Antibody	Cat No.	Manufacturer	Application
TRIM47	26885-1-AP	Proteintech	WB, IHC
HNF4α	ab181604	Abcam	WB, IP, IHC
HNF4α	PP-K9218-00	R & D Systems	WB, IHC
HNF4α	sc-374229	Santa Cruz Biotechnology	IP
IgG	A7028	Beyotime	WB
Flag	F7425	Sigma	WB
HA	H6908	Sigma	WB
V5	ab27671	Abcam	WB
Ub	3933	Cell Signaling Technology	WB
Ki-67	350513	Biolegend	ICFC
Ki-67	ab16667	Abclonal	IHC
β-Actin	A5441	Sigma-Aldrich	WB

Table S3 RNA interference.

Gene(human)	Primer (5′–3′)
siHNF4A	CCACAUGUACUCCUGCAGATT
siTRIM47-1	TGAAGCTCCCAGGGACTATTT
siTRIM47-2	TACTGGGAGGTGGAGATTATC

Table S4 Correlation between TRIM47 expression and clinicopathologic parameters in HCC patients.

Characteristics	TRIM47-High n=41	TRIM47-Low n=41	P value*
Age, n (%)			0.432
≤60	30 (36.6%)	33 (40.2%)	
>60	11 (13.4%)	8 (9.8%)	
Gender, n (%)			0.331
Female	4 (4.9%)	7 (8.5%)	
Male	37 (45.1%)	34 (41.5%)	
HBV infection, n (%)			0.067
No	13 (15.9%)	6 (7.3%)	
Yes	28 (34.1%)	35 (42.7%)	
AFP(ug/L), n (%)			0.185
>400	24 (29.3%)	18 (22%)	
≤400	17 (20.7%)	23 (28%)	
Tumor size(cm), n (%)			0.173
>5	35 (42.7%)	30 (36.6%)	
≤5	6 (7.3%)	11 (13.4%)	
Tumor capsule, n (%)			0.034
Yes	23 (28%)	32 (39%)	
No	18 (22%)	9 (11%)	
PVTT, n (%)			0.359
No	24 (29.3%)	28 (34.1%)	
Yes	17 (20.7%)	13 (15.9%)	
CEA(ug/L), n (%)			0.140
≤5	31 (37.8%)	36 (43.9%)	
>5	10 (12.2%)	4 (4.9%)	
≤6	0 (0%)	1 (1.2%)	
Recurrence, n (%)			< 0.001
No	14 (17.1%)	31 (37.8%)	
Yes	27 (32.9%)	10 (12.2%)	
Metastasis, n (%)			0.085
No	26 (31.7%)	33 (40.2%)	
Yes	15 (18.3%)	8 (9.8%)	
Pathologic.stage, n (%)			0.778
Stage III	36 (43.9%)	34 (41.5%)	
Stage II	4 (4.9%)	5 (6.1%)	
Stage IV	1 (1.2%)	2 (2.4%)	
TNM Stage, n (%)			0.817
I	15 (18.3%)	14 (17.1%)	

Characteristics	TRIM47-High n=41	TRIM47-Low n=41	P value*
II+III	26 (31.7%)	27 (32.9%)	
BCLC Stage, n (%)			0.111
A+B	22 (26.8%)	29 (35.4%)	
C	19 (23.2%)	12 (14.6%)	

The median value of all 82 HCC samples was chosen as the cut-off point separating HCC patients with high TRIM47 expression and low TRIM47 expression.

MVI microvascular invasion, PVTT portal vein tumor thrombus, BCLC Barcelona Clinic Liver Cancer staging. *Chi-square test.

2. Supporting figures

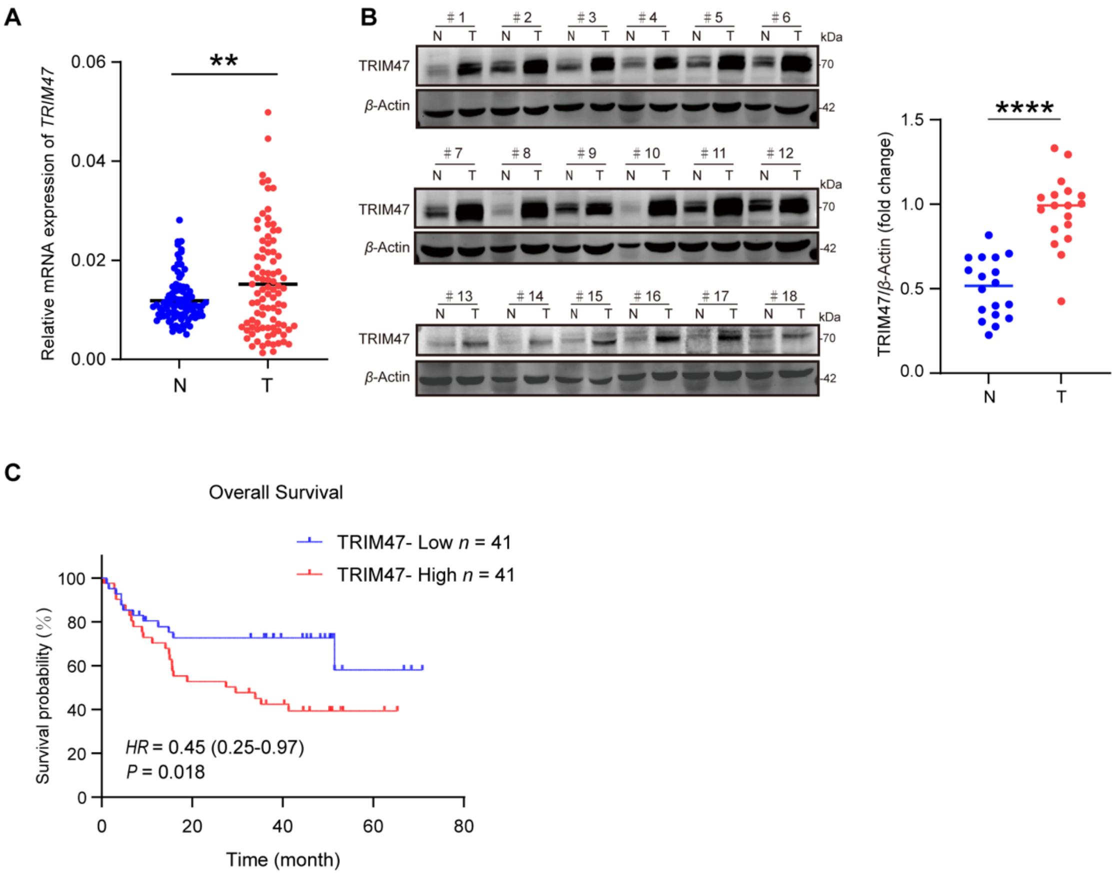

Figure S1 Elevated TRIM47 expression correlates with poorer prognosis in HCC. (A) qRT-PCR analyses of *TRIM47* mRNA in paired tumor tissues (T) and adjacent non-tumor liver tissues (N) from 82 HCC patients. (B) Semi-quantification of TRIM47 protein expression detected by Western blotting analysis in paired tumor tissues (T) and adjacent non-tumor liver tissues (N) from 18 HCC patients. (C) Overall survival (OS) of 82 HCC patients with low (n = 41) or high (n = 41) *TRIM47* mRNA expression, based on the median value as the cut-off, was analyzed using the log-rank test. Statistical analyses were performed using two-tailed Student's t-test (A, B) or Kaplan–Meier analysis/Gehan–Breslow–Wilcoxon test (C). Data are presented as mean ± SEM for (A, B). **P<0.01, ****P<0.0001.

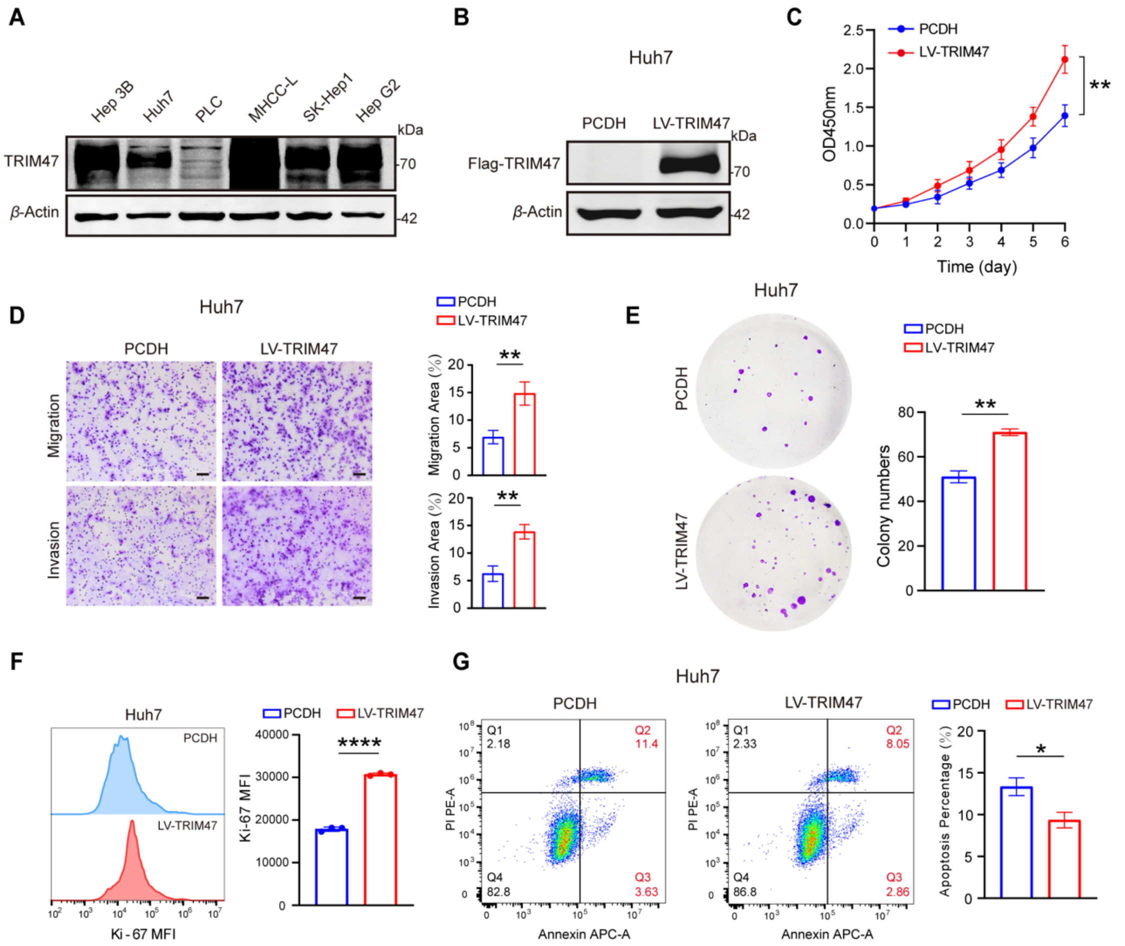

Figure S2 TRIM47 overexpression promotes malignant characteristics in Huh7 cells. (A) Western blotting analysis of TRIM47 protein levels in six HCC lines. (B) Western blotting analysis of TRIM47 protein levels in Huh7 cells infected with lentivirus expressing TRIM47 (LV-TRIM47) or the control lentivirus (PCDH). (C) The proliferation of Huh7 cells infected with LV-TRIM47 or PCDH was detected using CCK8 assays. (D) Migration (top) and invasion (bottom) assays of Huh7 cells infected with LV-TRIM47 or PCDH. Scale bar, 100 μm. (E) Representative images (left) of colony formation assays and the statistical results (right) of Huh7 cells infected with LV-TRIM47 or PCDH. (F) Mean fluorescence intensity of Ki-67 in Huh7 cells infected with lentivirus expressing TRIM47 (LV-TRIM47) or the control lentivirus (PCDH). (G) Apoptosis was assessed in Huh7 cells infected with LV-TRIM47 or PCDH using Annexin V/PI staining. Statistical analyses were performed using two-tailed Student's *t*-test (E–G) or two-way ANOVA with multiple comparisons (C). Data are presented as mean ± SD for (C, E, F, G). *P<0.05, **P<0.01, ****P<0.0001; ns, not significant.

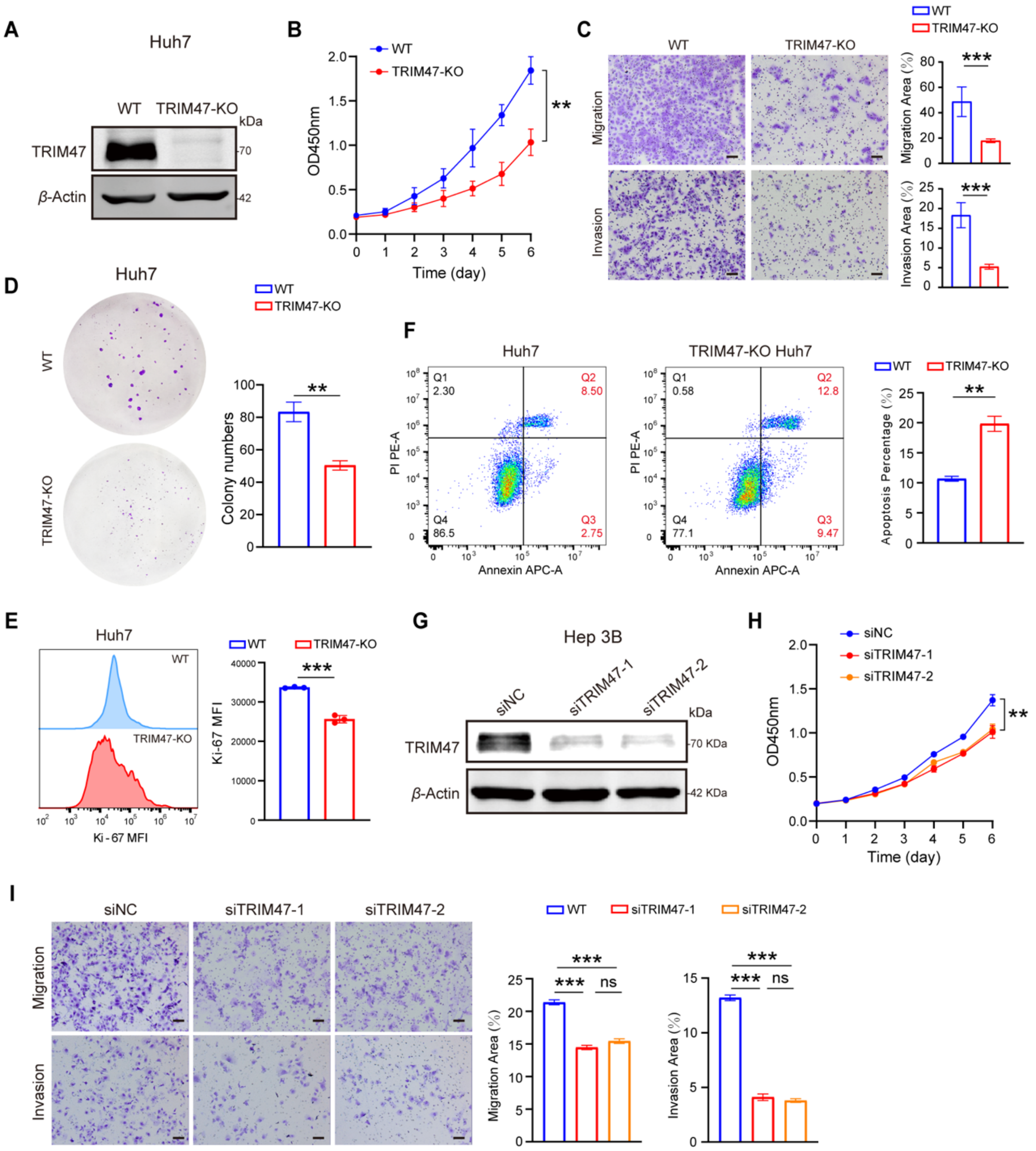

Figure S3 TRIM47 knockout or knockdown suppresses malignant phenotypes in HCC cells. (A) Western blotting analysis of TRIM47 protein levels in wild-type (WT) and TRIM47 knockout (TRIM47-KO) Huh7 cells. (B) The proliferation of wild-type and TRIM47-KO Huh7 cells (CCK-8 assays). (C) Transwell assays showed reduced migration and invasion in TRIM47-KO Huh7 cells compared to wild-type (WT) Huh7 cells. Scale bar, 100 μm. (D) Representative images (left) of colony formation assays and the statistical results (right) for wild-type and TRIM47-KO Huh7 cells. (E) Mean fluorescence intensity of Ki-67 in wild-type and TRIM47-KO Huh7 cells. (F) Apoptosis in WT and TRIM47-KO Huh7 cells was quantified by Annexin V/PI staining. (G) Western blotting analysis of TRIM47 protein levels in siNC and knockdown Hep3B

cells. (H) The proliferation of siNC- and TRIM47-KD-treated Hep3B cells (CCK-8 assays). (I) TRIM47 knockdown in Hep3B cells suppressed migration and invasion capacities compared to the siRNA controls (siNC) in transwell assays. Scale bar, 100 μm. Statistical analyses were performed using two-tailed Student's *t*-test (C, D, E, F, I) or two-way ANOVA with multiple comparisons (B, H). All data are presented as mean ± SD. ***P*<0.01, ****P*<0.001; ns, not significant.

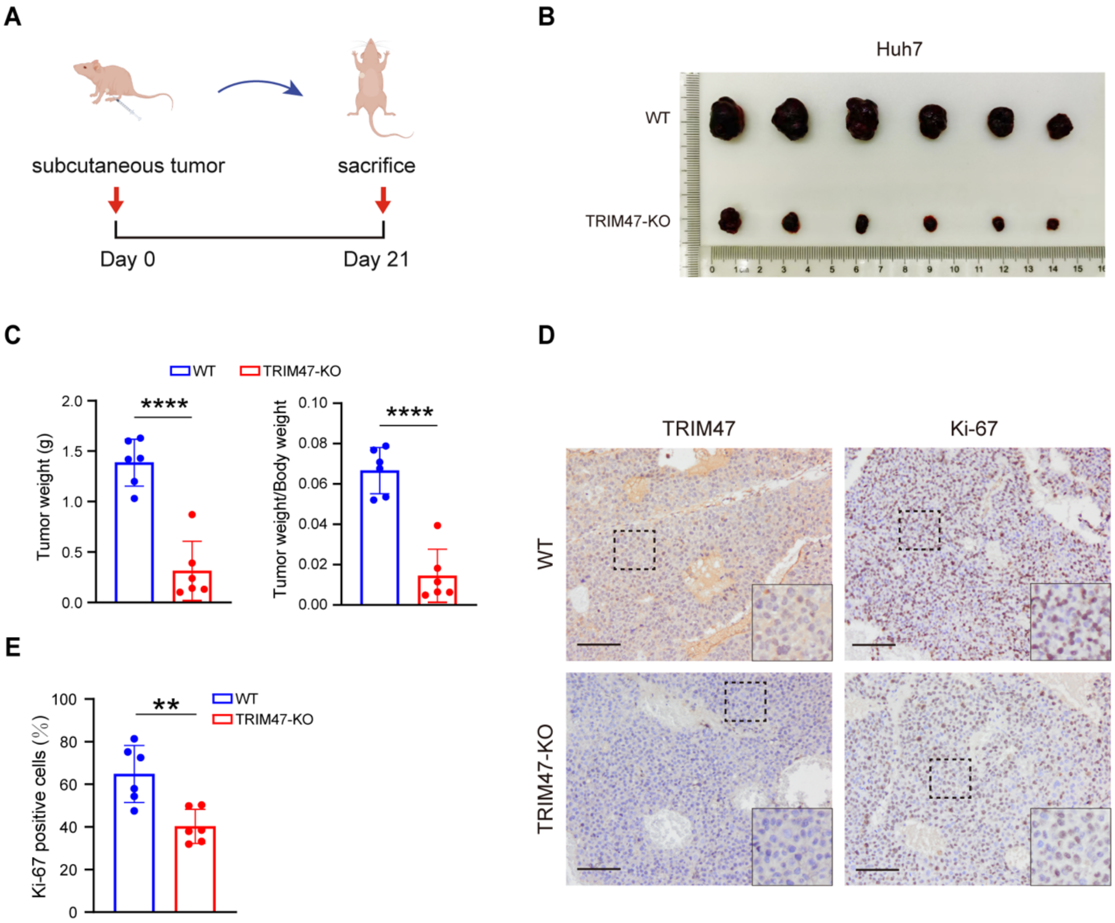

Figure S4 *TRIM47* knockout suppressed subcutaneous tumor growth of HCC *in vivo*. (A) Schematic diagram of the experimental procedures for xenograft HCC models using wild-type or TRIM47-KO Huh7 cells (2 × 10^6 cells suspended in 100 μL DMEM). (B) Images of xenograft tumors from mice inoculated with wild-type or TRIM47-KO Huh7 cells after 21 days. (C) Statistical results for wild-type and TRIM47-KO tumor xenografts. (D) Representative images of IHC staining for TRIM47 and Ki-67 in tumor xenografts. Scale bar, 100 μm. (E) Quantitative analysis of Ki-67-positive areas in transplanted tumors. Statistical analyses were performed using a two-tailed Student's *t*-test (C, E). All data are presented as the mean ± SEM. ***P*<0.01, *****P*<0.0001.

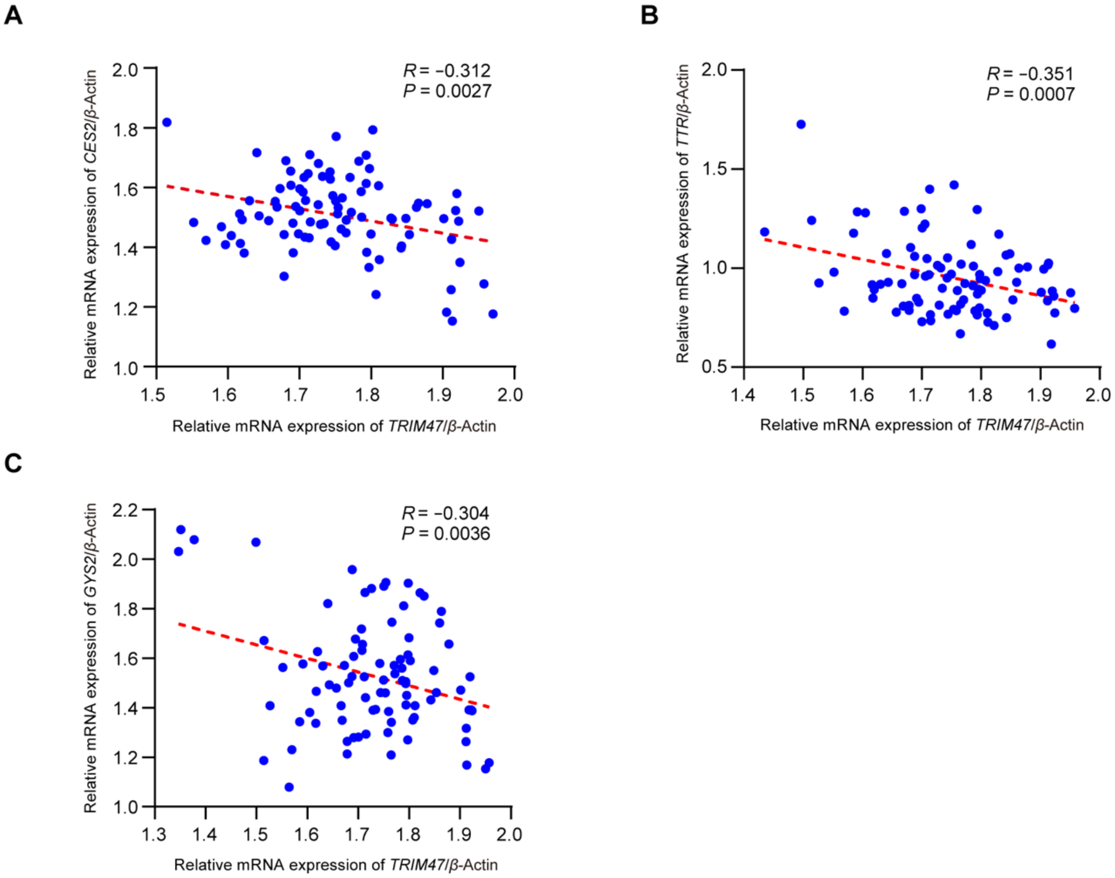

Figure S5 Correlation analysis between *TRIM47* and *HNF4A* target gene in 90 human specimens. (A–C) Correlation analysis of *TRIM47* mRNA expression with *CES2* (A), *TTR* (B), and *GYS2* (C) gene expression levels in 90 HCC tissues revealed statistically significant associations in all three pairs. Statistical analyses were performed using two-tailed Spearman's correlation test (A–C).

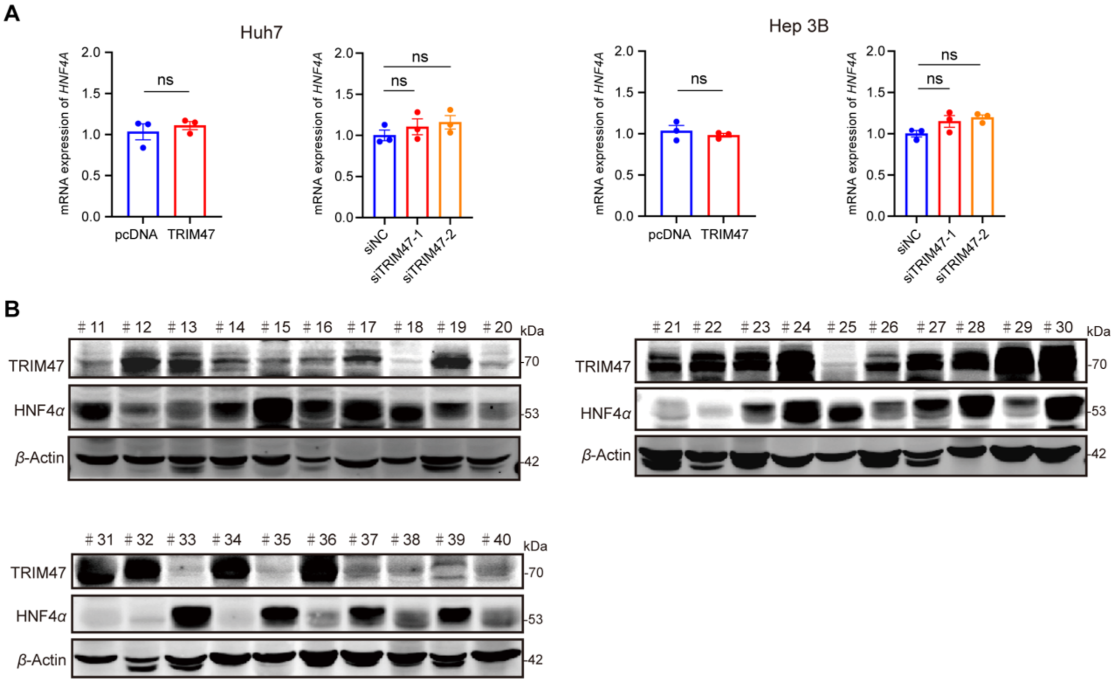

Figure S6 TRIM47 suppresses HNF4*α* levels in HCC. (A) Relative mRNA expression of *HNF4A* in Huh7 and Hep3B cells transfected with either pcDNA3.0 or Flag-TRIM47 plasmids, or with siNC or siTRIM47. (B) Western blotting analysis of HNF4*α* and TRIM47 protein levels in human HCC tissues. Statistical analyses were performed using a two-tailed Student's *t*-test(A). All data are presented as mean ± SD. ns, not significant.

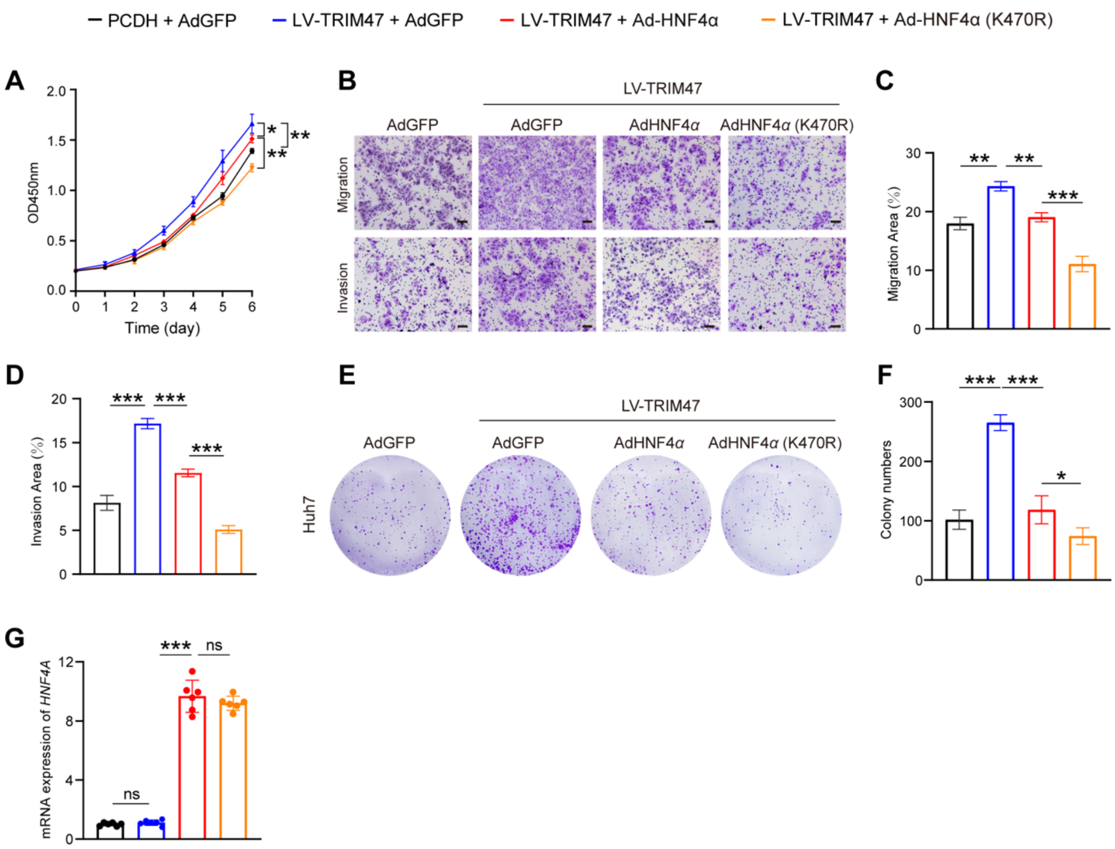

Figure S7 The HNF4*α*-K470R mutant inhibits TRIM47's ability to promote cell proliferation *in vitro*. (A) CCK-8 assays of Huh7 cells infected with control lentivirus (PCDH) or lentivirus TRIM47 (LV-TRIM47), together with adenovirus expressing GFP, HNF4*α*, or HNF4*α*-K470R. (B–D) Migration (top) and invasion (bottom) assays of Huh7 cells infected with LV-TRIM47 or PCDH, along with adenovirus expressing GFP, HNF4*α*, or HNF4*α*-K470R (B). Scale bar, 100 μm. Statistical results of migration (C) and invasion (D). (E, F) Representative images of colony formation assays (E) and statistical results (F) of Huh7 cells infected with LV-TRIM47 or PCDH, together with adenovirus expressing GFP, HNF4*α*, or HNF4*α*-K470R. (G) *HNF4A* mRNA levels in xenograft tumors of the indicated groups (n = 6). Statistical analyses were performed using two-tailed Student's t-test (C, D, F, G) or two-way ANOVA with multiple comparisons (A). Data are presented as mean ± SD (A, C, D, F) or the mean ± SEM (G). *P<0.05, **P<0.01, ***P<0.001; ns, not significant.

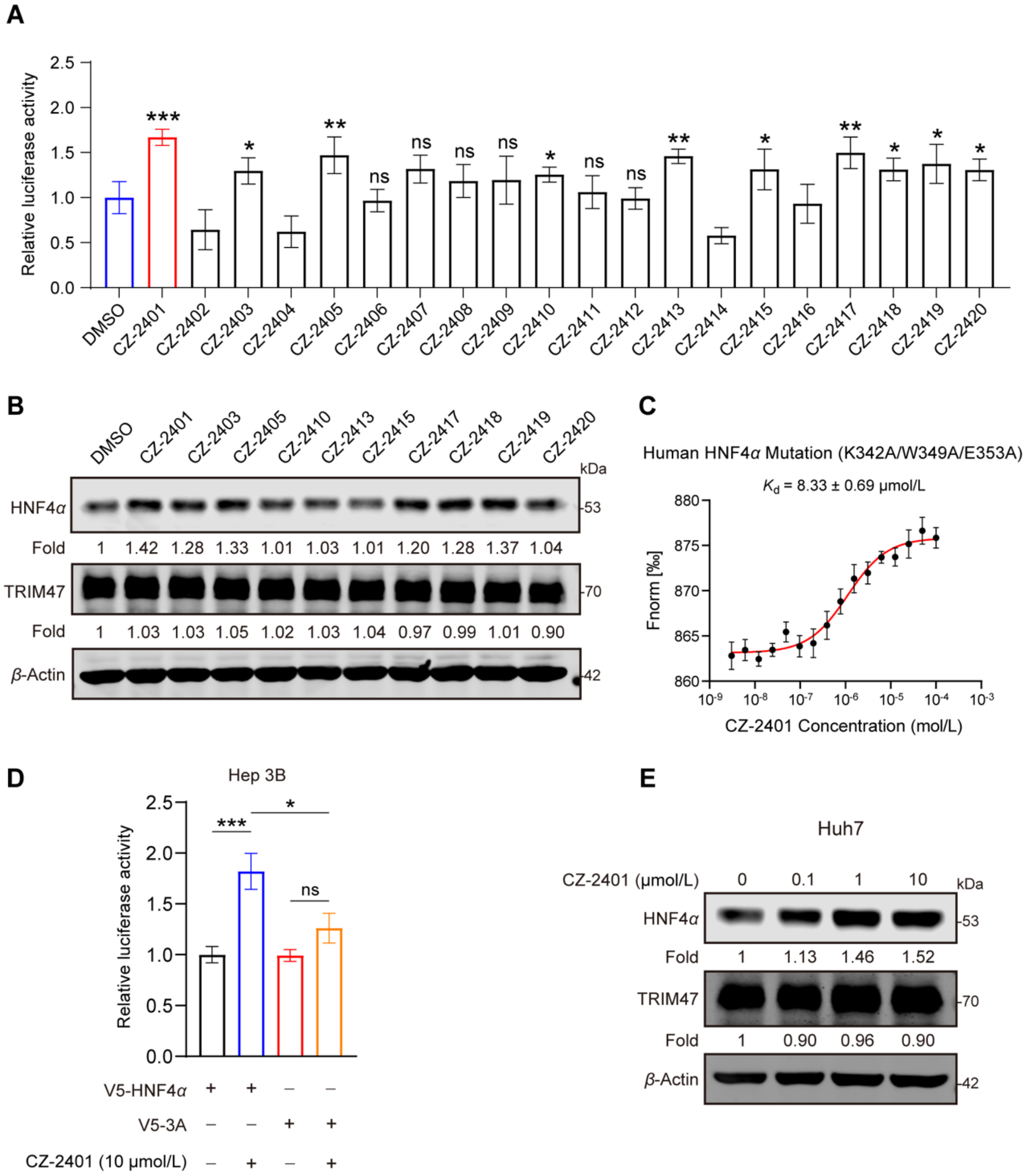

Figure S8 Screening of small-molecule compounds. (A) Hep3B cells were treated with 20 small-molecule compounds selected from preliminary screening, at a concentration of 10 μmol/L for 24 h. The transcriptional activity of HNF4*α* was then assessed using a reporter gene assay. (B) Western blotting analysis showed HNF4*α* protein levels of Hep3B cells treated with the compounds (10 μmol/L) enhanced HNF4*α* transcriptional activity for 24 h. Fold changes calculated relative to DMSO-treated controls (set as 1). (C) MST assay detecting the binding of CZ-2401 to HNF4*α* (K342A/W349A/E353A) mutation *in vitro*. (D) The relative luciferase reporter activity of HNF4*α* and HNF4*α* (3A) mutation in Hep3B cells treated with CZ-2401 (10 μmol/L) for 24 h. (E) Western

blotting analysis of HNF4α in Huh7 cells treated with different concentrations of CZ-2401 for 24 h. The vehicle control was adjusted to 1. Statistical analyses were performed using a two-tailed Student's *t*-test (A, D). All data are presented as mean ± SD. *P<0.05, **P<0.01, ***P<0.001; ns, not significant.

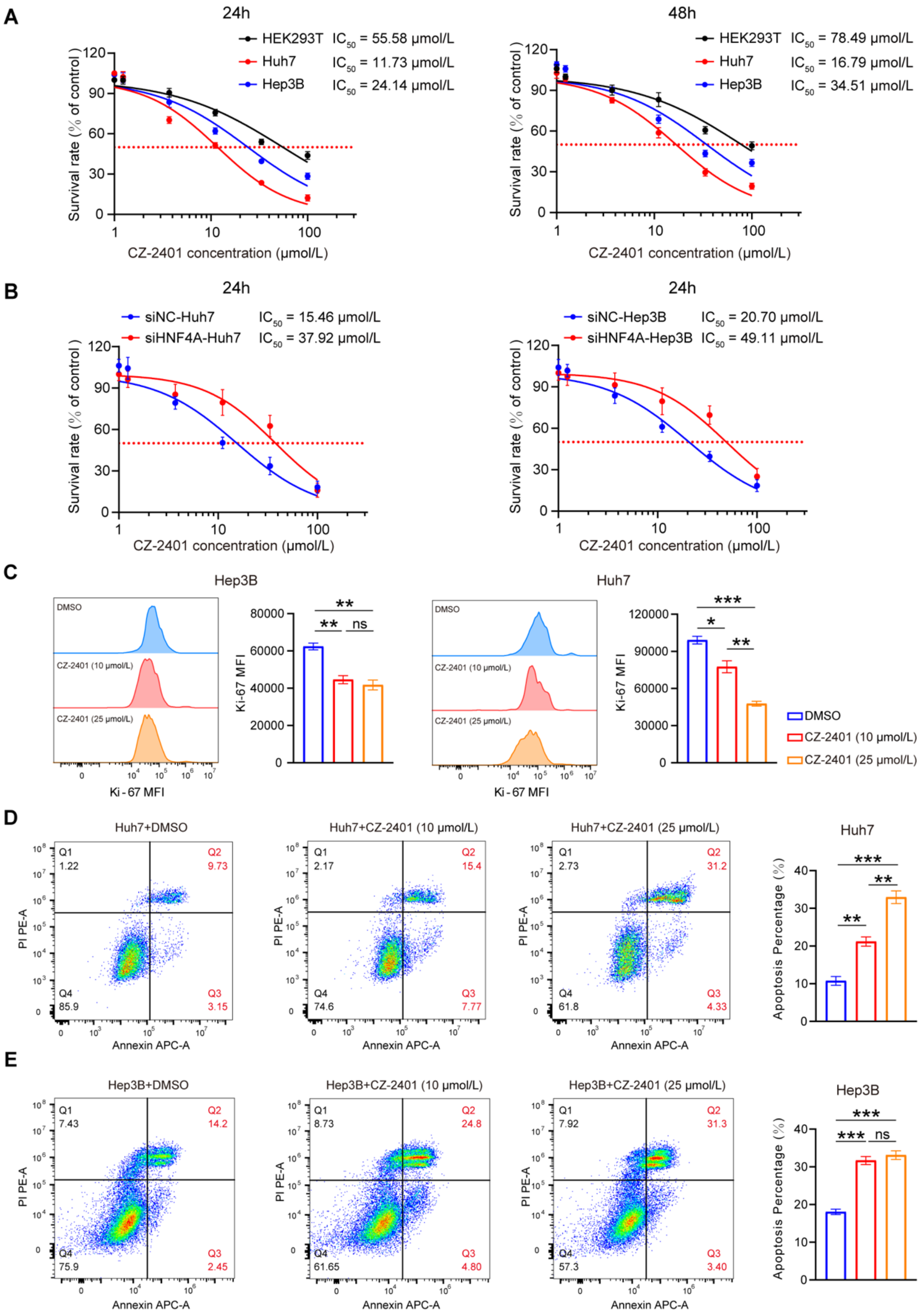

Figure S9 CZ-2401 enhances the stability of HNF4α protein. (A) Cytotoxicity assays were conducted in HEK293T, Huh7, and Hep3B cells to assess the general safety profile of CZ-2401. (B) Huh7 and Hep3B cells were transfected with siNC and siHNF4A for 24 h, followed by treatment with increasing concentrations of CZ-2401

for 24 h to assess cytotoxicity. (C) Mean fluorescence intensity of Ki-67 in Huh7 cells treated with CZ-2401 for 24 h. (D, E) Apoptosis was assessed in Huh7 (D) and Hep3B (E) cells treated with CZ-2401 for 24 h using Annexin V/PI staining. Statistical analyses were performed using two-tailed Student's *t*-test (C, D, E). All data are presented as mean ± SD. *P<0.05, **P<0.01, ***P<0.001; ns, not significant.
